# ‘I Get High With a Little Help From My Friends’ - How Raves Can Invoke Identity Fusion and Lasting Co-operation via Transformative Experiences

**DOI:** 10.3389/fpsyg.2021.719596

**Published:** 2021-09-24

**Authors:** Martha Newson, Ragini Khurana, Freya Cazorla, Valerie van Mulukom

**Affiliations:** ^1^School of Anthropology and Conservation, University of Kent, Canterbury, United Kingdom; ^2^Centre for the Study of Social Cohesion, University of Oxford, Oxford, United Kingdom; ^3^Department of Sociology, University of Warwick, Coventry, United Kingdom; ^4^Centre for Trust, Peace and Social Relations, Coventry University, Coventry, United Kingdom

**Keywords:** psychedelics, identity fusion, rave, awe experiences, transformative experiences, social bonding, 4Ds, dance

## Abstract

Psychoactive drugs have been central to many human group rituals throughout modern human evolution. Despite such experiences often being inherently social, bonding and associated prosocial behaviors have rarely been empirically tested as an outcome. Here we investigate a novel measure of the mechanisms that generate altered states of consciousness during group rituals, the 4Ds: **d**ance, **d**rums, sleep **d**eprivation, and **d**rugs. We conducted a retrospective online survey examining experiences at a highly ritualized cultural phenomenon where drug use is relatively uninhibited- raves and illegal free parties. Engaging in the 4Ds at raves or free parties was associated with personal transformation for those who experienced the event as awe-inspiring, especially for people with open personalities (*n* = 481). Without awe, or a ritual context, indulging in the 4Ds was associated with a lack of personal growth, or *anomie*. A complex SEM revealed that personal transformation following awe-inspiring raves was associated with bonding to other ravers and prosocial behavior toward this group at a cost to self in a simple economic game. Bonding to humanity was not associated with these events. The findings suggest that employing the 4Ds in a ritualized environment - particularly dancing and drug use – can help build meaningful social bonds with associated positive behavioral outcomes.

## Introduction

Humans have come together for exalted, communal experiences for more than 100,000 years (Ember and Carolus, 2017). These occasions – both ancient and current - often involve repetitive drums, dance, sleep deprivation and, often, psychedelic substances. Their cumulative effect on the group results in a sense of coming together, termed ‘*collective effervescence’* (Durkheim, 1915) or ‘*communitas’* (Turner, 1969). Indeed, one of the main consequences of such collective gatherings is considered a unification of participants, or social bonding (Block, 2020; Savage et al., 2020). Here we examine a novel measure for the mechanisms driving altered states of consciousness during awe-inspiring group experiences in rave culture, a modern cultural phenomenon that has been likened to entheogenic shamanic practices (Tramacchi, 2000; St John, 2015). This measure brings together the 4Ds: **d**ancing, **d**rums, sleep **d**eprivation, and **d**rugs. We address a current gap in the literature around psychedelic drug consumption during culturally organic, awe-inspiring rituals and their relation to transformative experiences, social bonding, and prosocial behavior. We also explore the unique role psychedelic drugs play in this process, compared to other popular recreational drugs that invoke feelings of connectedness, such as MDMA.

One aspect of an awe-inspiring event that might help individuals to experience the group as bigger than its individual parts is *liminality* (Van Gennep, 1960; Turner, 1969). Liminality describes a withdrawal from the culturally normal modes of social action, in which ego may be dissolved and new perspectives gained. This ‘betwixt and between’ state (Turner, 1969) facilitates *communitas* by homogenizing secular distinctions like rank or status, instead replacing them with intense comradeship and egalitarianism. One particularly potent form of social bonding, *identity fusion*, concerning a visceral oneness to the group (Swann et al., 2009) has been described as a socio-cognitive product of *communitas* both theoretically (Whitehouse and Lanman, 2014) and empirically (Páez et al., 2015; Kettner et al., 2021). Here, in a natural setting, we investigate the precursors to awe-inspiring, liminoid experiences, and their relationships with social bonding and prosocial behavior.

Ritual liminality can be induced by several identifiable human behaviors, including what we term ‘the 4D’s’, which are: (1) **d**ancing (Lewis-Williams, 1992); (2) intense rhythmic **d**rums (Savage et al., 2020); (3) sleep **d**eprivation (Dahl, 2013); and (4) **d**rugs, especially psychedelics (Hood, 2014). This suite of behaviors is powerful enough to alter our state of consciousness and in so doing take a group far away from its conventional realms of normality (or the profane), and into the surreal and sacred. Importantly, this liminal state gives group members an opportunity to transcend the boundaries between self and group. Psychedelic drugs have long played a role in ritual and shamanic ceremonies; indeed, human serotonergic receptors are unusually receptive to psychedelic binding compared to other primates (Pregenzer et al., 1997), which has been used as evidence for the role of psychedelics in human evolution (Winkelman, 2017). Nonetheless, it remains unclear just how important psychedelics are in relation to the other elements of the 4Ds, especially in relation to intergroup dynamics and pro-social behaviors within the group.

Here, we first introduce the 4Ds in the research context of rave culture, before discussing the role psychedelics play in ego dissolution and feelings of awe (Millière, 2017; Nour and Carhart-Harris, 2017; van Mulukom et al., 2020). Next, we explain how sharing a transformative experience with other group members can lead to an intense form of group bonding, i.e., identity fusion (Newson et al., 2016; Buhrmester et al., 2018), and how this may be related to awe-inspiring experiences during raves.

### Altered States of Consciousness and the 4Ds

The 4Ds are a novel approach to capture the mechanisms for altered states of consciousness in natural settings, which we investigate here for the first time. The 4Ds include four main ritual features: **d**ance, **d**rums, sleep **d**eprivation, and **d**rug consumption. Each element of the 4Ds alone can have a powerful effect on individual and collective consciousness and behavior, but when used together the effects have the potential to be culturally seismic. The 4Ds occur in varying formats cross-culturally but are broadly long-standing in modern human evolution. For instance, while rave culture may use electronic music and chemically synthesized psychedelic drugs to reach a desired altered state of consciousness (St John, 2004), indigenous Amazonian cultures use rhythmic singing and drumming with ayahuasca (Metzner, 2014).

First, dancing has been described as religious by its very nature (Gauthier, 2004) and it is dance which constitutes the architecture of a rave. Dance fills the void of formal structure - no tables, no chairs - just a space constructed by raving bodies, formed simultaneously by free agents and a merged, united mass. Csikszentmihalyi(1975:43) listed dance as a form of play that is intrinsically simulating because it enacts a holistic total body involvement. He termed this ‘flow’. Dance as flow merges the dancer with the act resulting in loss of identity and a fusion with the wider world, an experience that can be identified in the popular expression ‘I lost myself in the music’. These sentiments are doubtlessly found cross-culturally, from the dancing of Egyptian Sufis (St John, 2004) to the medicinal trance-dance of the !Kung (Lewis-Williams, 1992). Indeed, dance is ubiquitous across human societies, perhaps partly because the synchronous element of dance produces endorphins, which are central to human bonding merge (Tarr et al., 2014; Fink et al., 2021).

Second, and intricately connected to dancing, are ‘drums’ or intense, repetitive beats, which have long been associated with altered states of consciousness (Winkelman, 1986). The psycho-spiritual benefits of drumming are said to include physical and spiritual healing (Aldridge and Fachner, 2006). Significant decreases in cortisol, a key stress hormone, have been found after just 15 minutes of listening to drumming in a controlled experiment (Gingras et al., 2014). The Dobe Ju| ’hoansi (a tribe in modern day Botswana and Namibia) achieve altered states of consciousness through rhythmic beats, all night dancing and flickering lights – techniques which are also used at raves (Hutson, 2000:39; Lee, 1967). Although the DJ has been likened to a shaman (Gerard, 2004), the free party scene has an emphasis on dancers rather than DJ, a feature cited as revolutionary for its move away from 400 years of bourgeoisie-led music (Gauthier, 2004). In Durkheimian terms, this small, temporary, and self-organizing society can self-worship using a stack of speakers for its totem.

Third, sleep deprivation alters brain activation (Gujar et al., 2010) and is linked to disassociation (Selvi et al., 2015). Despite the hegemonic Northern-European/American preference for a single block of uninterrupted nocturnal sleep, the effects of sleep deprivation are not universally perceived as wholly negative. For instance, the ‘vision quests’ associated with delirium induced by sleep-deprivation are actively sought out in some cultures, such as some Native American communities (Dahl, 2013), and many others that practice shamanism (Winkelman, 1990). Raves and free parties primarily feature in the night-time economy, though they may continue for several days, so sleep deprivation tends to be a prerequisite for participation. Drugs may further disturb sleep patterns. For instance, ayahuasca, although commonly consumed nocturnally, alters sleep patterns even when clinically administered in the daytime (Barbanoj et al., 2008). The fourth and final D, drugs are perhaps the most obvious candidate for altering states of consciousness for their potentially rapid and intense changes to perception and behavior (Tart, 1969; Tramacchi, 2000; Preller et al., 2019).

### Psychedelics and Ego Dissolution

While the positive long term therapeutic effects of psychedelics have been relatively well attended to since the resurgence of clinical psychedelic research (Rougemont-Bücking et al., 2019), their role in intergroup dynamics is less understood. Psychedelics, serotonergic hallucinogens, induce an altered state of consciousness commonly framed as powerful modifications to perception and mood lasting several minutes to 24 hours, or even more, depending on what is consumed, how much, and the method of ingestion. CSPs (classically serotonergic psychedelics) are generally safe and, unlike many other recreationally used drugs, are not associated with addiction (Nichols, 2016). Psychedelics include naturally occurring substrates, such as psilocybin, ayahuasca, peyote, and mescaline and chemically made drugs including LSD ((5*R*,8*R*)-(+)-lysergic acid-*N,N*-diethylamide) or synthetic DMT. In all cases, these psychedelics work on serotonin 5-HT_2__A_ (5-hydroxytryptamine 2A) receptors (Nichols, 2016).

Psychedelic experiences are often characterized by a reduced sense of the self (Yaden et al., 2017), or even more extreme, ego dissolution, whereby the boundaries of the ego are temporarily disrupted (Millière, 2017). “Drug-induced ego dissolution”, where the boundary between self and world becomes blurred, has been proposed as a strong candidate for the potentially life-changing or spiritual experiences that psychedelics are reported to induce (Millière, 2017; Nour and Carhart-Harris, 2017). During certain types of ego dissolution, the self can be felt to be integrated into a greater whole, and an increased feeling of unity with one’s surroundings is experienced (Nour et al., 2016).

However, recent research has demonstrated that psychedelic experiences involving strong feelings of awe, but not necessarily ego dissolution, are associated with increased levels of feeling bonded with humanity and nature up to five years after the event (van Mulukom et al., 2020). Awe promotes the ‘small self’ and plays a significant role in prosocial behaviors (Piff et al., 2015). As such, psychedelic-inspired awe may play a key role in psychedelic therapies (Hendricks, 2018). Importantly, van Mulukom et al. (2020) found that the effect of awe on bonding to humanity and nature was driven by feelings of connectedness (as a subcomponent of awe) during the psychedelic experience, rather than by feelings of self-diminishment or perceived vastness. The study did not measure feeling bonded with psychedelic-taking co-participants however, which begs the question of the more immediate bonds psychedelic drugs can induce.

Another drug regularly taken during raves, MDMA (3,4-Methylenedioxy methamphetamine), can also have dissociative and/or depersonalizing effects. Often classed as an ‘empathogen’, rather than a psychedelic, it is more popular though less associated with ‘mystical’ experiences (Lyvers and Meester, 2012). The altered state of consciousness associated with MDMA does not appear to be associated with the 5-HT_2__A_ receptor (Puxty et al., 2017). Instead, increased heart rate associated with a moderate dose of MDMA in clinical settings has been associated with disassociation. Psychedelics consumed at mass festivals have been associated with transformative experiences more than MDMA (Forstmann et al., 2020).

Of the ‘big five’ personality traits (neuroticism, extroversion, openness, agreeableness, and conscientiousness), openness has a special relationship to psychedelics (Nichols, 2016). Previous research has demonstrated a positive association between the openness personality trait and total number of psychedelic experiences, specifically in that psychedelic use is believed to modulate personality (Erritzoe et al., 2019). Furthermore, openness has been associated with serotonergic neurotransmission but not frontal serotonin-2A-receptor binding, i.e., psychedelic rather than MDMA use is particularly related to the openness trait (Erritzoe et al., 2019). Research using double-blind trials suggests that psychedelics lead to lasting augmented trait openness - particularly mystical experiences have been linked to significant increases in otherwise stable trait openness a year later (MaClean et al., 2011). While psychedelics may increase openness to experience, openness may also influence the extent to which a psychedelic experience is perceived as more or less awe-inspiring (Yaden et al., 2019) and, consequently, how personally transformative that experience is. Here, we examine how trait openness relates to psychedelic ritual experiences (i.e., the 4Ds in the context of raves or free parties) and awe.

### Transformative Experiences and Social Bonding

Previous research suggests that experiencing highly emotional, momentous events together creates a particularly strong and visceral type of group bonding called *identity fusion* (Swann et al., 2009, 2012). This fusion of personal and group identities is often compared to another, weaker form of group bonding termed *identification* (Whitehouse and Lanman, 2014). In relation to Durkheim’s *collective effervescence*, identity fusion has been likened to organic solidarity – an evolutionary old form of group alignment that kept small groups tightly bound for hunting big game or defending one another in tribal warfare (Whitehouse et al., 2017). Identification, in contrast, anonymizes larger, agricultural groups for the purpose of swiftly recognizing group members who would otherwise be strangers if it were not for displays of their group membership (i.e., mechanical solidarity). The pictorial fusion scale, used in this study to operationalize group bonding, gives respondents the opportunity for complete immersion in their group (Swann et al., 2009), thus extending the popular Inclusion of Self in Other scale (Aron et al., 1992).

Three main causal pathways to fusion have been posited in recent years: shared experiences, shared biology, and shared ideology (Gómez et al., 2020). Regarding shared experiences, which we investigate here, identity fusion is argued to be a psychological mechanism evolved to hold groups together when individuals undergo intense and personally transformative experiences together (Whitehouse et al., 2017). Examples of emotionally intense, shared events that induce fusion include fighting on the frontline (Whitehouse and Lanman, 2014), experiencing a traumatic childbirth (Tasuji et al., 2020), and even watching one’s football team endure a harrowing defeat (Newson et al., 2016; Newson et al., 2021). What is common to these events is that they contain powerful, emotional experiences that are felt to be personally transformative (Newson et al., 2016), and which are perceived to be similarly transformative for fellow participants (Buhrmester et al., 2018; Whitehouse, 2018).

In the psychedelics literature, personally transformative experiences have also been termed ‘quantum change’ or ‘self-transcendent experiences’ (Forstmann et al., 2020). Of these, epistemically transformative experiences are so intensely profound that they cause a shift in our values, attitudes, and sense of self in lasting and usually meaningful ways. Such experiences are equivalent to the personally transformative events described in the fusion literature, in that they are powerful enough to effectively re-write one’s sense of self to incorporate the group with which one experiences the said event. By a process of reflection (Jong et al., 2015) and a false consensus bias, whereby we believe others to have perceived the event in the same way as us (Ross et al., 1977), individuals who have a personally transformative experience may irrevocably fuse with the group (Whitehouse, 2018).

Indeed, Forstmann et al. (2020) found that transformative experiences and social connectedness both increased after recent use of psychedelics at a series of field studies at mass multi-day gatherings. Furthermore, social connectedness (measured using the inclusion of self in other or IOS scale) following psychedelic use increased via transformativeness. Reports of feeling more connected to humanity among users of psychedelics (Carhart-Harris et al., 2018; van Mulukom et al., 2020) may represent the projection of relational ties on to a larger social group, which is one of the premises of fusion theory (Swann et al., 2012). Recent research has connected identity fusion to the *communitas* felt by participants at arranged ceremonial psychedelic retreats, but the relationship between the actual drugs consumed and fusion was not analyzed (Kettner et al., 2021).

### Illegal Raves and Free Parties

Transcending to the sacred plane via awe-inspiring or liminal group experiences has been common in religious rituals for millennia (Dunbar, 2013), but this mechanism is also exploited by the secular, for instance at large music gatherings (Turner, 1969) or raves (Gauthier, 2004). Of these collective rituals, rave is a relatively recent, but seemingly enduring, subcultural phenomenon. The exhaustive all-night (or weekend-long) dancing, engulfing repetitive bass beats, and, often heavy psychedelic drug use coupled with little sleep, construct a potent liminoid space with the potential to generate intense feelings of awe and social bonds, establishing a collective effervescence unparalleled for many young initiates (Gauthier, 2004; St John, 2015).

In the UK, multiple public order and licensing acts have tried to curb illegal raves. Of particular interest to this research are ‘free parties’; raves that occur on the periphery of society and aspire to non-commercialization in contrast to the multi-million-dollar industry of the clubbing scene. This long-lasting counterculture evolved from the ‘free festival’ movement of the 1960s and 1970s (Griffin et al., 2018) and is often associated with subversive politics and drug use. With the emergence of mass raves and the popularization of electronic dance music (EDM) in the late 1980s and 1990s came a cultural split - the mainstream ‘rave’ and club scene on the one hand, and the ‘free party’ scene on the other.

In contrast to more mainstream clubs and music festivals, the liminoid spaces free parties and uncommercial raves occupy offer a greater opportunity for decreased self-regulation (Griffin et al., 2018) and, potentially more feelings of awe. They occur without security, or with underground security firms, un-ticketed or alternative payment systems (e.g., trade and exchange), and no licensing or insurance regulations. As the name implies, the individual has an opportunity to be ‘free’, whatever that may mean to them at that moment in time (Jaimangal-Jones et al., 2010). In contrast, music festivals offer a more bounded place for leisure or downtime. For an extended review of how raves constitute liminoid spaces, please see Supplementary Material 1.

Via liminality, rave culture accesses a ‘sacred’ realm in which ravers may have feelings of awe and experience *communitas*, in an engagement familiar to ritual devotees all over the world. Due to its transgressive nature, rave has been likened to a new religious movement, comparable to world entheogenic rituals (St John, 2004). This renders the rave an ideal context to test the effects of the ‘4D’s’ on awe and personal transformation and how, in turn, these effects of rave attendance may lead to social bonding and prosocial behaviors. As yet, partly due to the challenging nature of recruitment with underground subcultures, rave contexts have not been examined in terms of mechanisms that induce altered states of consciousness, nor has the specific role of psychedelic drugs.

We address these questions using an online survey with a natural population where ecological validity for psychedelic consumption is high compared to laboratory or clinical settings. Compared to the current proliferation of Northern-hemisphere targeted ayahuasca or psilocybin retreats that borrow heavily from indigenous cultures and make up much of the naturalistic psychedelic research, rave is an organic cultural practice. Drug taking is not prescriptive or particularly guided at these events, and usage varies between participants, making it a viable candidate for experiences with a natural control population of non-drug using participants.

### Present Study

So far, research into the socially cohesive benefits of psychedelics beyond clinical or laboratory settings has been lacking (Millière et al., 2018). We help to address this issue by conducting a retrospective online survey in a natural setting (*n* = 481) with a hard to access population of self-identified ‘ravers’ who attended legal and illegal raves or ‘free parties’. In this study, through a set of pre-registered hypotheses, we examine the pathway from engaging in behaviors that alter states of consciousness to awe, which in turn are associated with personally transformative experiences, ultimately leading to group bonding and prosocial behavior in rave contexts (see Table 1).

**TABLE 1:** Overview of pre-registered hypotheses of the current study.

Nr.	Hypothesis
1a	Engaging in behaviors to alter states of consciousness at raves (i.e., the 4Ds) will be associated with awe, which in turn will be associated with personally transformative experiences.
1b	The effect engaging in the 4Ds has on awe is moderated by the openness personality trait: the more open a person is (traitwise), the more these behaviors contribute to awe experienced during the rave.
1c	Of the 4Ds - extended dancing, drums, sleep deprivation, and drugs - drug use will be most associated with awe, and more specifically, psychedelic drugs.
1d	Of the awe experiences, feelings of connectedness and self-diminishment will have the strongest association with personal transformativeness, over perceived vastness, altered time perception, physical sensations, and need for accommodation.
2a	Awe experienced during the rave will be associated with greater bonding to other ravers and humanity, when the rave was a personally transformative event.
2b	The effect of personal transformativeness on bonding to other ravers is moderated by sharedness, or how shared the rave participants consider their experience to be.
3	Personally transformative raves will have prosocial effects (measured in charity donations) when individuals feel most connected to the target group. Ravers will donate to rave-based/humanitarian charities, when they feel connected to other ravers/all of humanity, and will donate less when they report weaker connections to these targets.

First, we investigated whether participants engaged in the 4Ds (dance, drums, sleep deprivation, and drugs) at particularly memorable raves or free parties and whether this in turn was associated with feelings of awe and personal transformation (H1a). We focused on the roles trait openness (H1b) and ego-dissolving drugs (CSPs, psychedelics) played in the process (H1c). We also tested which types of awe experiences had the strongest association with personal transformativeness (H1d).

Next, we investigated how awe-inspiring raves might be associated with greater bonding to other ravers – or even to humanity – when that rave was a personally transformative event (H2a). We also tested whether a sense of having shared the experience with others might influence this process (H2b). Finally, we investigated prosociality using a simple economic game, and its links to both bonding and transformative experiences (H3).

In a series of exploratory analyses, we also explored self-reported past donations to these charitable targets, as well as the association between time elapsed since the memorable rave and charitable giving. As illegal raves occur at the periphery of society and are more likely to be implicated in illegal drug use, we explored differences between legal raves and illegal raves or free parties, including whether the latter generate more awe and, consequently more bonding. Finally, we checked for gender differences in our key variables.

## Materials and Methods

This study was pre-registered prior to data collection. The pre-registration, and the data and analytic code, are available on OSF.

### Participants

Ravers (*n* = 552) were recruited via adverts on specialized Reddit threads, dedicated rave Facebook groups, and via DJs on Instagram, calling for people aged 18 and over who had been to raves or free parties in the last five years. There were 16 participants who failed to complete two awareness checks and were excluded from the dataset. We further excluded 39 participants who reported having attended a rave more than 5 years ago, 3 who reported never having been to a rave, and 28 who completed less than 45% of the survey, leaving *n* = 481 (see Table 2 for descriptives). Those who completed at least 45% of the survey completed everything required for this particular study. Further questions pertained to rave attendance and wellbeing during lockdown, for a separate paper.

**TABLE 2:** Descriptive statistics for key variables.

Demographics			

Age		Ethnicity	
Mean (*SD*)	30.51 (9.27)	Asian	32.10%
Range	18-68	White	28.10%
		Black	22%
**Gender**		Mixed	9.80%
Women	54.70%	Other	6.40%
Men	43.20%	Chose not to say	2.30%
Chose not to say	2.10%		

**Rave/free party attendance**			

**Awe-inspiring event**		**Lifetime**	

Illegal	28.30%	Ever attended illegal	53%
People in attendance*	300 (6-80,000)	Total attended*	10 (1-2000)
Took drugs	44.7%		
*Alcohol*	54.7%		
*MDMA*	34.3%		
*Cannabis*	22.0%		
*Cocaine or other stimulants*	17.1%		
*Psychedelic drugs*	14.1%		
*LSD*	6.9%		
*Mushrooms*	3.7%		
*DMT or others from the 2C-x family*	2.9%		
*Other hallucinogens*	0.6%		
*Ketamine*	13.9%		
*Other drugs (e.g., self-reports of coffee or laughing gas)*	2.5%		
*Other opioids*	0.4%		
*Heroin*	0.0%		

**Social bonding**			

**Other ravers**		**Humanity**	

Fused (i.e., option E)	15.6%	Fused (i.e., option E)	17.0%
Previously donated to a rave charity		Previously donated to a humanitarian charity	
*Never*	87.5%	*Never*	28.7%
*Once or twice*	7.4%	*Once or twice*	32.1%
*Sometimes*	2.5%	*Sometimes*	22.6%
*Quite often*	1.1%	*Quite often*	10.0%
*Regularly*	1.5%	*Regularly*	6.6%

*median (range).

### Recruitment and Sample Size

Recruitment was via snowball sampling. A broad range of rave backgrounds were targeted using multiple Reddit subforums and Facebook specialist groups (including techno, psytrance, drum and bass, traveling communities, psychedelics groups, and the clubbing scene). After two weeks we had only attained half of our intended participants, so we created a pre-screening survey on Prolific to recruit a similar demographic. Snowball-sampled participants (38.7%) were incentivized with the chance to win a £100 prize (1 in 100 chance) and Prolific participants (61.3%) were remunerated for their time (£9.50 p/h). Data were collected in February - March 2021 via a Qualtrics survey. The snowball sample scored significantly higher on several central measures, including transformativeness and awe, compared to the Prolific sample, so we also re-ran the main analyses whilst including recruitment as a covariate and found them to be robust against recruitment (see Supplementary Material 2).

We conducted a power analysis with the R package semPower. Our goal is to obtain an RMSEA value of.06 (Kline, 2011), with a standard.05 alpha error probability and 1 - beta value of 0.95 (see R script line below) for our largest model (see Exploratory Analyses; df = 17; all variables included and one interaction). The power analysis returned indicated that we would need N = 477 participants for a RMSEA fit of.06. R code: semPower.*aPriori*(effect = 0.06, effect.measure = “RMSEA”, alpha = 0.05, beta = 0.05, df = 17).

### Measures

First, we asked participants to describe a rave or free party that they felt was particularly profound or awe-inspiring. To control for memory effects, we requested that they recall an event from the last five years. They also stated what year the event happened, roughly how many people were there, how many raves or free parties they had been to in total, and whether they had been to an illegal rave or free party. We used a figure reported in the BBC that the largest rave ever held in the UK comprised 20,000 ravers and used that as a cut off for outliers.

Personal transformativeness was measured using items that draw from work by Newson et al. (2016) and Buhrmester et al. (2018) on a −3 - + 3 Likert scale (SD - SA), including: The event has shaped me as a person; The event has personally transformed me; I am who I am today because of this event.

The 4Ds were designed to assess rave-specific altered states of consciousness. These were measured with two items each on a 1-7 Likert thermometer (SD - SA) and were presented in a random order. Dance: *I danced constantly; I didn’t dance at all* (RC). Drums: *At times, the music was so intense it was all I could hear; The music wasn’t loud at all* (RC). Sleep Deprivation: *I have rarely slept so little; I had my normal amount of sleep* (RC). Drugs: *By my standards, I was very high on drugs; I was completely sober* (RC). The 4Ds were further assessed with additional items on a 1-70 thermometer type scale, but these had poor fit, potentially due to the quite different scales they used, so were excluded from the final scale. These items can be found in Supplementary Material 2.

Participants reported whether they had taken any drugs during the experience (see Table 2 for a breakdown). From these answers (e.g., alcohol, cannabis, cocaine, crack, other stimulants, LSD/acid, magic mushrooms, DMT, 2CB or others in this family, other hallucinogens, heroin, other opioids, other), a binary psychedelics variable and a binary MDMA variable were computed

Awe was measured using the AWE-S scale (Yaden et al., 2019), measured on a −2 to + 2 scale (SD vs. SA). The scale includes six subscales (self-diminishment, connectedness, time perception, perceived vastness, physical sensation, and need for accommodation). Please see SI for items. We included two moderators: openness and sharedness. Openness was measured using the BFI subscale on a −2 to 2 scale (SD-SA) (John et al., 1991).

How shared the participant felt the experience to be with other individuals was measured using a scale based on Muzzulini et al. (2021) on a 1-7 scale (‘not at all’ to ‘a lot’), including the following three items: *Consider the rave experience you described and rate the extent to which other people at that event: (1) would give the exact same answer as you; (2) share the same feelings about that event as you; (3) share the same memories about that event as you.*

Social bonding was measured using Swann et al. (2009)’s 5-point pictorial identity fusion scale, which was treated continuously for model analyses given a relatively normal distribution. Identity fusion was measured with reference to ‘other people who have been to raves/free parties’ and ‘all of humanity’.

Prosocial behavior was measured using a simple economic game designed to test participants’ preference for self, a rave-based ingroup, or an extended ingroup (humanity) by inviting them to split an imaginary £10 as they wished between the three options. First, participants read a few sentences about each charity, which were taken from the websites of the respective charities but presented anonymously to prevent factors such as nationalism unduly influencing the results. The charities were Psycare UK and Médecins Sans Frontières (MSF). Participants were told that at the end of the study, the researchers would donate £100 to the charity that received the largest amount of imaginary donations, and that if most people chose to keep the money for themselves, then we would not give a donation to charity. We donated £100 to MSF. The order in which the charity texts were presented was randomized. We also asked participants if they had ever given to similar charities in the past.

Demographic information was asked at the end of the survey, including age, gender, and ethnicity (white, Asian, black, mixed, other, prefer not to say).

### Statistical Analyses

We computed means for the personal transformativeness, sharedness, AWE-S, and Openness scales. We also computed means for dancing, drumming, drugs, and sleep deprivation, as well as a 4D mean, which is the mean value of the four 4D scales. All variables were standardized before entering them in mediation/structural equation models and regression models. We used the standard *p* < 0.05 threshold for determining if our results were significantly different from those expected if the null hypothesis were correct. For the overall SEM model (see exploratory analysis), we considered RMSEA ≤ 0.08, combined with CFI > 0.90 and SRMR ≤ 0.08, to be indicative of an acceptable model fit (Kline, 2011). If a subject did not complete parts of a scale that were required to compute a mean or a total, then their responses to that measure were excluded from analysis. Participants were expected to answer all questions due to forced logic in the survey design. All analyses were conducted in R (R Team, 2013), using the lme4 package for linear regressions (Bates et al., 2007), lavaan for mediation modeling (Rosseel, 2012), and ggplot2 for plotting figures (Wickham, 2011). The default estimator of the lavaan package (which is maximum likelihood) was selected in path analyses as we did not have prior hypotheses about alternative estimation methods.

#### Pre-registered Hypotheses

To test H1a we conducted a mediation model examining the effect of the 4Ds (X) on personal transformativeness (Y) via awe (M). To test H1b, we conducted a moderated mediation model examining the moderated effect of the 4Ds (X) on personal transformativeness (Y) via awe (M), moderated by openness (mod). To test H1c, we conducted a regression model including the 4Ds (IVs) and awe (DV). To test H1d, we conducted a regression model including all awe experiences (IVs) and personal transformativeness (DV). To test H2a, we conducted a mediation model examining the effect of awe (X) on bonding to target groups (Y) via personal transformativeness (M). To test H2b, we conducted a moderated mediation model examining the effect of awe (X) on bonding to target groups (Y) via personal transformativeness (M), which was moderated by perceived sharedness of the experience (mod). To test H3, we conducted a mediation model examining the effect of personal transformativeness (X) on charity donations to a rave or humanitarian charity (Y) via bonding to other ravers/all of humanity (M).

#### Exploratory Analyses

To explore the effects of classic serotonergic psychedelic drugs (CSP; cf. van Mulukom et al., 2020), we entered psychedelic use as an additional predictor in the regression predicting awe as a result of the 4Ds. In the same regression, we also explored the effect of MDMA and combinations of CSP drugs and MDMA, given the prevalence of this drug at rave parties, and its effect on group bonding.

**FIGURE 1:**
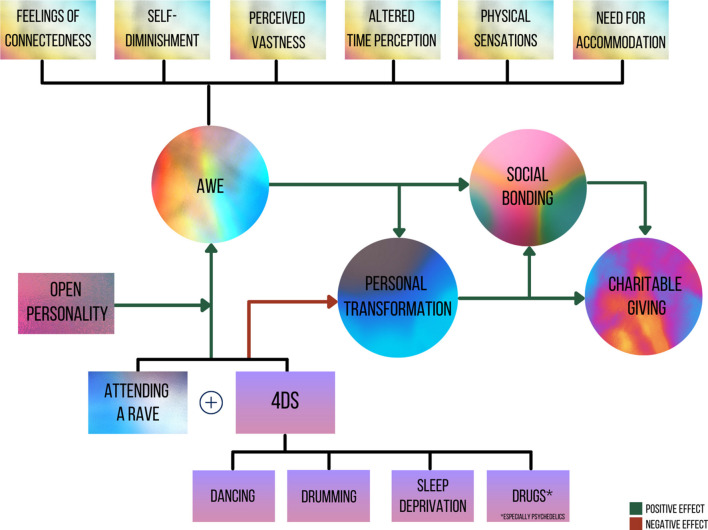
Full model from the 4Ds to prosocial behaviors via awe, transformativeness, and social bonding.

We also made a pre-registered prediction under exploratory analyses that self-reported past, as well as imagined, donations to the humanitarian and rave charities would be significantly predicted by social bonding. To explore this, we replaced the imagined donation DVs in the model from H3 with the past donation DVs. Finally, we combined the mediation models from the pre-registered hypotheses together in one model (Figure 1). To achieve this, we added three extra direct paths to account for covariance: from 4Ds (X) to Bonding to target group (Y), from 4Ds (X) to Charity donation (Y), and from Awe (X) to Charity donation (Y).

In addition to our pre-registered hypotheses, we explored whether legal raves were quantifiably different from illegal raves or free parties, by comparing the key variables in our main model by event type using a series of Bonferroni-corrected independent t-tests (or Welch tests where equal variance was not assumed) and chi-squared tests. We also compared men and women’s responses to key variables using the same approach. A correlation matrix can be found in Supplementary Material 4.

## Results

### Pre-registered Hypothesis Testing

#### The 4Ds Lead to Personally Transformative Experiences via Awe, Especially for Particularly Open People

**FIGURE 2:**
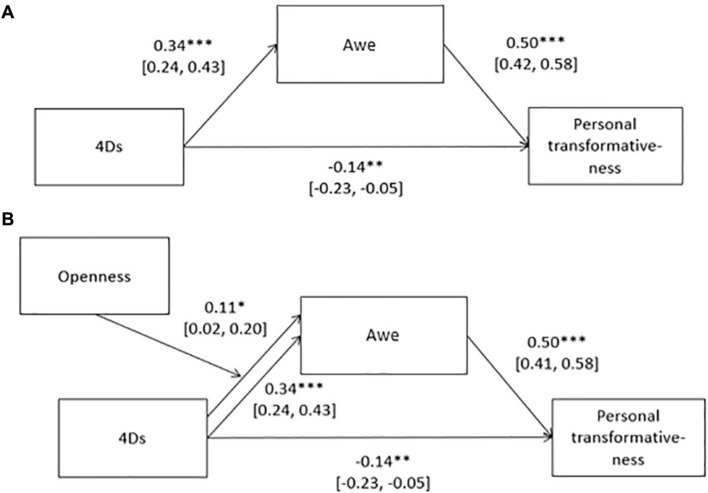
Mediation models testing the effect of the 4Ds on personal transformativeness via awe; **(A)** without moderator of openness **(B)** with moderator. Standardized beta coefficients with 95% CIs.^∗^*p* < 0.05, ^∗∗^*p* < 0.001, ^∗∗∗^*p* < 0.001.

We found support for the hypothesis that engaging in behaviors to alter states of consciousness at raves (i.e., having a score for the 4Ds) induces awe, which in turn is associated with personally transformative experiences (H1a), see Figure 2 for direct effects (see Supplementary Material 5 for full statistics). The indirect path from the 4Ds to personal transformativeness via awe was significant (*b* = 0.17 [0.12, 0.23], *p* < 0.001), though the total path was not (*b* = 0.03 [−0.08, 0.12], *p* = 0.57), indicating that the effect of the 4Ds on transformativeness was fully mediated by awe. We also found support for the hypothesis that the more open a person is (traitwise), the more engaging in the 4Ds contributes to awe experienced during the rave (H1b). The indirect path from the 4Ds to personal transformativeness via a moderated awe path was also significant (*b* = 0.05 [0.01, 0.10], *p* < 0.001), though the indirect path without moderation was significantly stronger (*p* = 0.001). When adding recruitment type as a covariate, the indirect path became non-significant (*b* = 0.09 [−0.01, 0.18]), though the mediator was still positively predicted (*b* = 0.10 [0.002, 0.195]).

Next, we investigated the unique roles of each of the 4Ds in relation to awe. While drugs had a strong effect on awe, dance was the largest predictor, followed by sleep deprivation, with no measured effect of more drums, Table 3. Given that the direct path from the 4Ds to personal transformativeness was significant, we also explored how the 4Ds were associated with reduced personal transformativeness through a regression including each of the 4Ds as predictors of personal transformativeness (see Supplementary Material 6 and Supplementary Table 5). We found that while dancing (β = 0.17 [0.08, 0.26], *p* < 0.001) and drug use (β = 0.12 [0.03, 0.22], *p* = 0.009) predicted increased transformativeness, drums significantly predicted reduced transformativeness (β = −0.20 [−0.30, −0.11], *p* < 0.001), with no significant direct effect of sleep deprivation (β = −0.05 [−0.15, 0.05], *p* = 0.30).

**TABLE 3:** Model for Hypothesis 1c: Predicting awe with the 4Ds separately.

Variable	β	SE	95% CI	*t*-value	*p*-value
Intercept	<−0.01	0.04	[−0.08, 0.08]	< 0.01	> 0.99
Dance	0.21***	0.05	[0.12, 0.30]	4.68	< 0.001
Drums	−0.02	0.05	[−0.11, 0.07]	–0.41	0.68
Deprivation	0.13**	0.05	[0.04, 0.22]	2.80	0.005
Drugs	0.18***	0.04	[0.09, 0.27]	4.04	< 0.001
Statistics	*F*(4,479) = 19.39, *p* < 0.001				
Fit	*R*^2^ = 0.139**, 95% CI[0.08, 0.19]				

All variables were standardized before they were added to the regression. *p < 0.05. **p < 0.01, ***p < 0.001.

**FIGURE 3:**
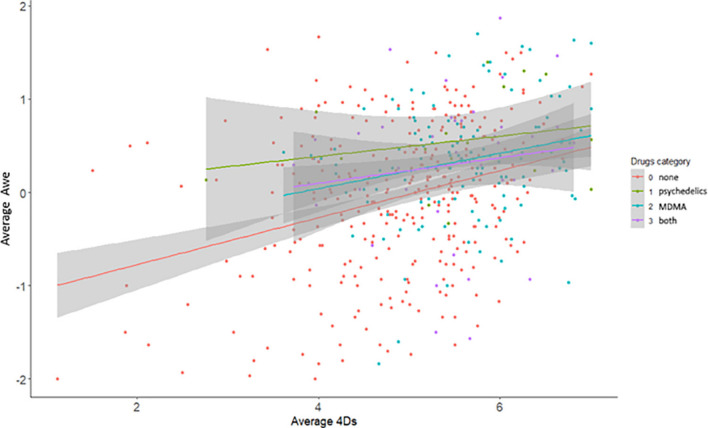
Scatterplots of the effect of the 4Ds on awe, grouped by drugs taken during the rave.

We also explored the differential effects of CSP drugs, MDMA, and the combination of MDMA and CSP drugs on awe. Taking different types of drugs had a significant effect on awe (*F*(3, 477) = 11.27, *p* < 0.001). Bonferroni-corrected *post hoc* comparisons demonstrated that those who took no drugs (*M* = −0.05, *SD* = 0.79) experienced less awe than those who took psychedelic drugs (*M* = 0.55, *SD* = 0.52), MDMA (M = 0.34, SD = 0.62), or both (M = 0.30, SD = 0.77). CSP drugs and MDMA both statistically contributed to awe and subsequent transformativeness, however psychedelic drugs had the greatest effect on awe, see Figure 3 (and Supplementary Material 6 with the full statistics). For all participants, the 4Ds together had the most significant effect on the awe path (β = 0.28 [0.20, 0.37], *p* < 0.001). However, the effect of taking (just) psychedelic drugs had the strongest effect on awe, compared to not taking any drugs at all (β = 0.59 [0.12, 1.07], *p* = 0.01), followed by taking MDMA (β = 0.30 [0.10, 0.51], *p* = 0.004). The effect of taking both psychedelic drugs and MDMA compared to not taking any drugs was not significant (β = 0.28 [−0.04, 0.59], *p* = 0.09).

#### Awe and Personal Transformation

**FIGURE 4:**
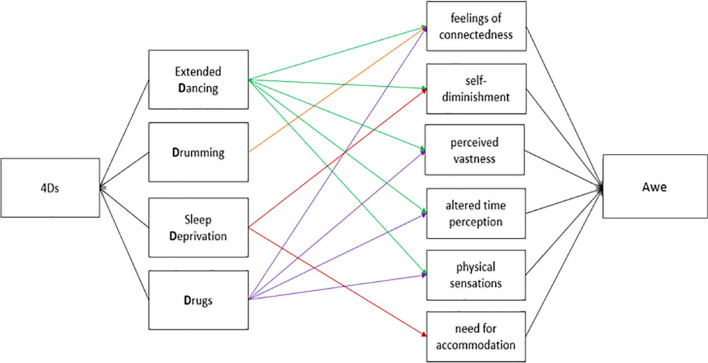
Separate regressions of each of the 4D components onto each of the awe components (colored lines); only significant relations are shown.

The hypothesis that of the awe sub-scales, feelings of connectedness and self-diminishment would have the strongest effect on personal transformativeness (H1d) was not supported. Instead, the perception of vastness, feelings of connectedness, and physical symptoms predicted transformativeness over the other awe subscales (Table 4). The positive effect of the 4Ds (the mechanisms that precede altered states of consciousness) on transformativeness via awe was driven by dance and drugs (Table 3 and Figure 4, and Supplementary Material 7).

**TABLE 4:** Model for Hypothesis 1d: Predicting transformativeness with the awe subscales.

Variable	β	SE	95% CI	*t*-value	*p*-value
Intercept	<−0.01	0.04	[−0.08, 0.07]	–0.09	0.93
Connection	0.21***	0.06	[0.09, 0.34]	3.33	< 0.001
Time perception	0.02	0.05	[−0.08, 0.12]	0.37	0.71
Vastness perception	0.23**	0.07	[0.09, 0.37]	3.17	0.002
Physical sensations	0.14**	0.05	[0.04, 0.25]	2.62	0.009
Accommodation	-0.06	0.05	[−0.16, 0.04]	–1.11	0.27
Self-diminishment	< 0.01	0.05	[−0.10, 0.10]	< 0.01	> 0.99
Statistics	*F*(6,475) = 27.38, *p* < 0.001				
Fit	*R*^2^ = 0.257**, 95% CI[0.19, 0.31]				

Note. All variables were standardized before they were added to the regression. *p < 0.05. **p < 0.01, ***p < 0.001.

#### Transformative Rave Experiences Lead to Bonding to Ravers, but Not Humanity

**FIGURE 5:**
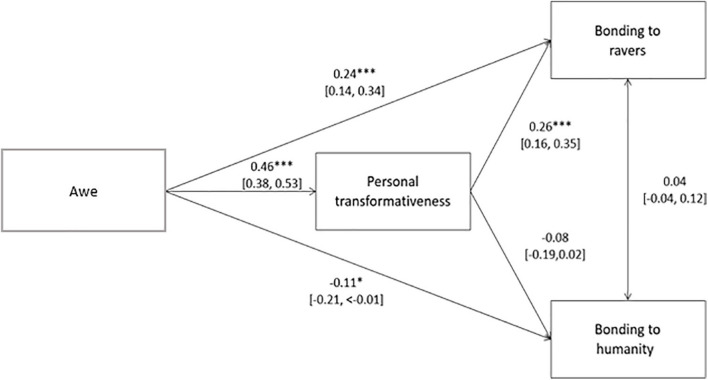
Mediation model predicting bonding to ravers and humanity from awe via personal transformativeness. Standardized beta coefficients with 95% CIs. ^∗^*p* < 0.05, ^∗∗^*p* < 0.001, ^∗∗∗^*p* < 0.001.

We found support for the hypothesis that awe experienced during a rave is associated with greater bonding to ravers when the rave was a personally transformative event (H2a), see Figure 5 for direct effects (and Supplementary Material 8 for full statistics of the model). Both the indirect path (*b* = 0.11 [0.07, 0.16], *p* < 0.001), from awe to bonding via personal transformativeness, and total path (*b* = 0.37 [0.29, 0.45], *p* < 0.001) were significant, suggesting a significant role of the direct path from awe to personal transformativeness. However, we did not find support for the hypothesis that the effect of personal transformativeness on bonding to other ravers was moderated by how shared ravers consider their experience to be (Supplementary Material 8). In the moderated mediation models, the indirect path *without* sharedness as a moderator (*b* = 0.11 [0.06, 0.16], *p* < 0.001) was significant, whereas the indirect path *with* the moderator (*b* < 0.01 [−0.04, 0.04], *p* = 0.84) was not. The two models were significantly different from each other (*p* < 0.001).

The second path in the model, from awe to bonding to humanity via personal transformativeness, was only partially, negatively supported (see Figure 5 above for direct effects, and Supplementary Table 8). The indirect path from awe to bonding to humanity via personal transformativeness was significant (*b* = −0.05 [−0.10, < −0.01], *p* = 0.03), as was the total path (*b* = −0.13 [−0.22, −0.04], *p* < 0.001). In the moderated mediation (see Supplementary Material 8), the indirect path *without* sharedness as moderator was significant (*b* = −0.05 [−0.10, <−0.01], *p* = 0.03), while the indirect path *with* the sharedness moderator was insignificant (*b* = 0.02 [−0.02, 0.06], *p* = 0.28), with a significant difference between the two indirect paths (*p* = 0.04). Given the significant direct paths from awe to bonding with ravers and bonding with humanity, we also explored which awe components predicted bonding to ravers and humanity, respectively, see Supplementary Table 15. We found that the direct path from awe to bonding with ravers was driven by positive associations with connectedness (*b* = 0.31 [0.19, 0.41], *p* < 0.001) and physical sensations (*b* = 0.22 [0.12, 0.33], *p* < 0.001), and negative associations with the need for accommodation (*b* = −0.24 [−0.35, −0.12], *p* < 0.001). The regression predicting bonding with humanity using the awe subscales was not significant (*F*(6,474) = 1.854, *p* = 0.09).

#### Bonding via Personally Transformative Raves Is Associated With Prosocial Behavior

**FIGURE 6:**
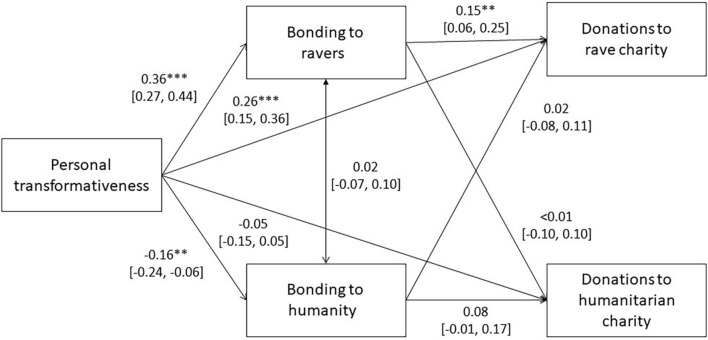
Mediation model of prosocial donations. Standardized beta coefficients with 95% CIs.^∗^*p* < 0.05, ^∗∗^*p* < 0.001, ^∗∗∗^*p* < 0.001.

Overall, participants donated more to the humanitarian charity (*M* = £4.49, *SD* = 3.40) than keeping money for themselves (*M* = £3.14, *SD* = 3.71; *p* = 0.001), both of which were allocated more money than the rave charity (*M* = £2.37, *SD* = 2.96; *p*’s < 0.004). Nonetheless, we found support for the hypothesis that personally transformative raves are associated with prosociality (measured in hypothetical charity donations) when individuals feel most bonded to the target group (H3, Figure 6).

Ravers donated to rave-based charities when they felt connected to other ravers and donated less when they reported weaker connections. Both the indirect path (*b* = 0.05 [0.02, 0.09], *p* = 0.002), from transformativeness to donations to a rave charity via bonding to other ravers, and total path (*b* = 0.31 [0.22, 0.40], *p* < 0.001) were significant, suggesting a significant role of the direct path from personal transformativeness to charity donations. However, we did not find support for the humanity model, in which both the indirect path and total path from transformativeness to donations to a humanitarian charity via bonding to other humanity were not significant (*b* < −0.01 [−0.02, 0.01], *p* = 0.14; *b* = −0.06 [−0.15, 0.03], *p* = 0.16).

We also further explored the same model, replacing choices in the simple economic game for actual past charitable giving to rave and humanitarian charities. Contrary to the prediction in our pre-registration, past donations to rave charities was not a significant outcome variable (indirect effect of transformativeness on past donations to a rave charity via bonding to ravers: *b* = 0.001 [−0.01, 0.01]), nor was past giving to humanitarian charities predicted by transformativeness via bonding to humanity (indirect effect: *b*** =** −0.001 [−0.01, 0.01]. However, logistic regressions showed that bonding to ravers significantly predicted past charitable giving to rave based charities (*b* = 0.38, *SE* = 0.14, *p* = 0.006). The estimated odds ratio demonstrated an increase of nearly 46% [Exp (*B*) = 1.46] for having donated to a rave charity for every one unit increase of bonding to ravers. In contrast, bonding to humanity was not found to contribute significantly to past giving to a humanitarian charity (*b* = 0.07, *SE* = 0.10, *p* = 0.47). We found that the more recent the memorable rave participants reported on, the more they gave to the rave charity (*r*(473) = −0.16, *p* = 0.001), but there was no correlation between time passed since the memorable rave and donations to the humanitarian charity (*p* = 0.132).

### Exploratory Analyses: 4Ds at Rave > Awe > Transformation > Bonding > Charitable Giving

**FIGURE 7:**
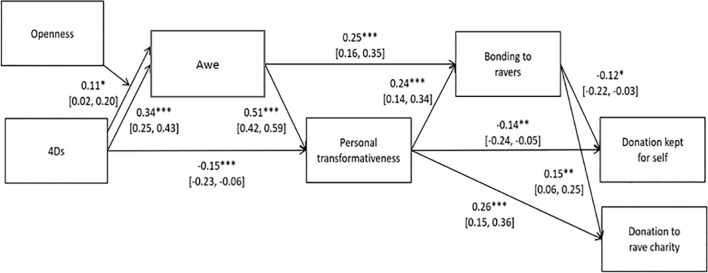
Full structural equation model of the 4Ds at raves > awe > transformativeness > bonding > prosociality pathway. Standardized beta coefficients with 95% CIs. ^∗^*p* < 0.05, ^∗∗^*p* < 0.001, ^∗∗∗^*p* < 0.001.

Finally, we combined the mediation models from the pre-registered hypotheses together in one model. The overall model (Figures 1, 7) incorporating the three mediation models had fit indices (RMSEA = 0.059; CFI = 0.969; SRMR = 0.040; see Supplementary Material 9 and Supplementary Table 16 for full statistics) within our pre-registered thresholds (RMSEA ≤ 0.08, combined with CFI > 0.90 and SRMR ≤ 0.08; Kline, 2011). This path model suggests that the 4Ds are associated with feelings of awe (especially for people with high trait openness), which in turn relate to personal transformation and a bonding to other ravers. Finally, this bonding, is associated with less selfish and more prosocial choices toward ravers in a simple economic game.

### Further Exploratory Analyses

Finally, we found that illegal raves or free parties differed from legal raves in several important ways (Table 5). Of the 4Ds, drug taking was unsurprisingly higher at illegal raves. All subsequent variables in the model were also significantly higher at illegal events (openness, awe, transformativeness, social bonding, and prosocial donations). Male participants reported significantly higher levels of trait openness and transformativeness than females (*p’s* < 0.004) but there were no other gender differences after Bonferroni corrections (see Supplementary Material 10).

**TABLE 5:** Comparing key variables between legal and illegal events.

Variable	Legality	*N*	*M (SD)*	*t*	*df*	*p*	95% CI of the difference
4Ds (Dance)	Legal	345	5.77 (1.29)	–1.891	306.35	0.060	−0.43; 0.01
	Illegal	136	5.98 (1.03)				
4Ds (Drums)	Legal	345	5.84 (1.26)	1.627	479	0.104	−0.04; 0.47
	Illegal	136	5.64 (1.34)				
4Ds (sleep Deprivation)	Legal	345	4.41 (1.66)	–1.51	479	0.132	−0.57; 0.07
	Illegal	136	4.65 (1.52)				
4Ds (Drugs)	Legal	345	4.19 (1.63)	–5.529	479	< 0.001*	−1.21; −0.57
	Illegal	136	5.08 (1.48)				
Awe	Legal	345	0.03 (0.75)	–3.391	479	0.001*	−0.41; −0.11
	Illegal	136	0.29 (0.77)				
Openness	Legal	345	0.68 (0.66)	–3.384	479	0.001*	−0.36; −0.10
	Illegal	136	0.92 (0.66)				
Transfor-mativeness	Legal	344	-0.49 (1.65)	–4.089	271.8	< 0.001*	−0.94; −0.33
	Illegal	135	0.15 (1.48)				
Bonding (ravers)	Legal	345	3.08 (1.16)	–3.705	479	< 0.001*	−0.66; −0.20
	Illegal	136	3.51 (1.10)				
Donation (rave)	Legal	341	2.026 (2.73)	–3.786	201.34	< 0.001*	−1.88; −0.59
	Illegal	132	3.27 (3.36)				

*Bonferroni-corrected significance, p < 0.005.

## Discussion

There is a long tradition of research on communal experiences that induce collective effervescence or *communitas*, such as those involving **d**ance, **d**rums, sleep **d**eprivation and/or **d**rugs (‘the 4Ds’). Here we tested a series of pre-registered hypotheses to investigate how these mechanisms might be associated with feelings of awe in the context of contemporary raves and free parties, and how such personally transformative experiences may lead to social bonding and prosocial behaviors.

First, the 4Ds appeared to be an effective measure of mechanisms for inducing altered states of consciousness and feelings of awe. In support of previous research feelings of awe were particularly strong for people with open personalities (Yaden et al., 2019). Here we connect that association with a higher score on the 4Ds. In the wider literature, psychedelic use has been found to increase trait openness (MaClean et al., 2011; Erritzoe et al., 2019), but here we identify the significant role this trait may play in allowing people to perceive an event that could otherwise be quite exhausting to be awe-inspiring and personally transformative. It is also possible, however, that people’s openness increased as a result of their experience with psychedelics, leading them to remember the event as more awe-inspiring and transformative. Experimental research is required to disambiguate these relationships.

Importantly, unless people reported elevated levels of awe, the 4Ds were negatively associated with personal transformation. This path of no personal growth could be considered a Durkheimian *anomie*. Presumably, ravers who dance to loud repetitive beats, stay up all night, and take excessive quantities of drugs without a feeling of awe, are quite simply exhausted. This is supported by exploratory regressions, which identify that loud or intense music (‘**d**rums’) was especially negatively associated with transformativeness. Relatedly, the neuroscience literature has shown how emotionally intense experiences are associated with amygdala activity, i.e., encoding and consolidation.

Psychedelic drugs were particularly associated with feelings of awe, compared to MDMA or drugs in general. Drugs, as well as Dance, had particularly strong positive effects on awe compared to Drums or sleep Deprivation in this context. However, it was the 4Ds taken together that had the strongest association with awe. In turn, of the five awe subscales we explored, perceptions of vastness, feelings of connectedness, and physical symptoms (e.g., having goosebumps) were most associated with transformativeness.

Awe experienced during a rave or free party - especially when illegal - appeared to take participants on a journey to irrevocable bonding with those around them. While previous research demonstrated a link between feelings of connectedness at the time of psychedelic experiences and bonding with nature and humanity up to five years later (van Mulukom et al., 2020), the present research included connectedness to one’s more immediate social circle (other ravers), as well as associated prosocial behaviors. Direct, positive paths from connectedness and physical sensations and direct, negative paths from need for accommodation to identity fusion with ravers were also found. This supports recent research showing that visceral (but not detailed) flashbulb memories concerning Brexit referendum results led to personal transformativeness via processes of reflection about the meaning of the event (Muzzulini et al., 2021). Memory viscerality also includes physical experiences (e.g., feeling tense all over) when recalling the event (Talarico and Rubin, 2003).

Identity fusion is thought to consist of a visceral feeling of oneness with a group, such as resulting from the sharing of highly emotional, often dysphoric, experiences (Whitehouse, 2018). It is unsurprising that the need for accommodation was negatively associated with transformativeness here: this AWE-S subscale consists of items regarding the unsolved nature of an experience, whereas transformativeness is arguably the result of meaning making and reflection on meaning, processes shown to be involved in identity fusion (Jong et al., 2015).

Although personal transformativeness’ role in identity fusion has now been well documented (Newson et al., 2016; Buhrmester et al., 2018; Whitehouse, 2018), the details about what makes some events more transformative than others has remained elusive. We propose that feelings of awe may inspire a ritual state of liminality, connecting participants to parts of themselves and one another that are inaccessible in day-to-day life. One way that Dance, Drums, and perhaps Drugs, may contribute to creating awe-inspiring, liminal experiences is by inducing synchrony between ritual participants.

Synchrony is a powerful mechanism for creating a feeling of unity (Tarr et al., 2014; Lang et al., 2017), a potentially important aspect of inducing awe. Drumming and dancing clearly have synchronous elements so future understandings of the 4Ds would do well to consider synchrony’s role in inducing awe in relation to the 4Ds. Furthermore, the distributed coordination observed in dancing associated with modern rave or club culture, i.e., patterns of coupled movement between individuals within a group rather than whole group unitary synchrony, has been linked to group affiliation in terms of both liking and conforming (Von Zimmermann et al., 2018). Variations in synchrony could also be a promising for further research in natural environments.

### Links to Prosociality

We found that imagined donations to an ingroup were associated with awe-inspiring, transformative experiences induced by psychedelic consumption at raves in a complex, pre-registered multi-mediation model. These findings corroborate previous research demonstrating that awe is associated with prosocial outcomes. For example, the induction of awe (through a video clip or recall) has been associated with increased prosocial behavioral intentions (of generosity, or to help a person in need). It has been suggested that such prosocial effects may occur via a perception of ‘small self’ that is associated with awe (; Shiota et al., 2007; Piff et al., 2015; Bai et al., 2017). More specifically, awe is considered to constitute “a shift in attention toward larger entities and diminishment of the individual self” (Piff et al., 2015, p. 884).

Some nuance may be required, however: While ego dissolution is typically equated with self-diminishment, we did not find a specific effect of self-diminishment on transformativeness and bonding, while we did find an effect of connectedness, in line with previous research (van Mulukom et al., 2020). While both ego dissolution and self-diminishment involve reduced focus on the self (e.g., resulting from the 4Ds), it may be that ego dissolution captures both self-diminishment and *self-expansion*. Rather than feel themselves ‘shrink’ (self-diminishment), participants’ decreasing self-focus may have instead felt like they were becoming part of something larger, whether abstract (e.g., nature or God) or a larger group. Indeed, dispositional awe-proneness is associated with self-concepts that include more statements about membership to very large categories (Shiota et al., 2007).

In the current study, prosocial donations – past or imagined - were only found to a rave-based charity, and not to a humanitarian-based charity. The value of the economic game in this context was that it could be manipulated to specific real-world scenarios (rave and humanitarian charities (Pisor et al., 2020)). Coupled with extensive ethnographies describing prosociality at, and following, raves, the measure seems relevant to test the theory (Tramacchi, 2000; St John, 2015).

Pro-social behavior in the economic game was linked to bonding and the personal transformativeness associated with particularly awe-inspiring raves where participants engaged in the 4Ds. Previous research has demonstrated that fusion is particularly aligned with extreme sacrificial behaviors, compared to more mild sacrificial behaviors (Paredes et al., 2020). If our prosocial outcome had been more extreme, e.g., protest or willingness to fight and die, the associations between fusion and behavioral choices may have been even stronger. Identity fusion and cocaine (an ego-inflating drug) have been found to interact among British football fans (Newson, 2021), such that highly fused fans who took more cocaine reported the most aggression toward their rivals. One explanation for the link is that cocaine-induced ego inflation leads to a heightened sense of agency and strength among highly fused people, who already feel imbued with their group’s strength (Swann et al., 2009, 2012). We found peaceful associations between fusion and psychedelic drug use: prosocial choices in an economic game, at a cost to self.

### Psychedelics and the Social Cure

The consequences of intense shared rituals - in terms of connectedness and prosocial behavior - have not previously been empirically tested in the context of raves or in relation to CSPs. A strength of the present research is that psychedelic use was reported at a culturally natural setting, i.e., organically occurring raves, as opposed to a commercial ‘retreat’. Millière et al. (2018) called for caution regarding the “problematic conflation of temporary states of self-loss with “selflessness” as a personal or social trait” (page 2). Our research offers a unique lens into the interpersonal nature of awe-based selflessness and connectedness to others. Furthermore, we offer an applied study that respects the value of ‘set and setting’ (Leary et al., 1963).

Our data, from a natural population, may also be regarded as tentative evidence of self-medication for the *communitas* and social connections the 4Ds, particularly psychedelic drugs, offer. This relates to the literature on improved social connection and wellbeing from expensive Westernized ayahuasca and similar retreats (e.g., Kettner et al., 2021). Future research could help to identify the features of an event that make it an awe-inspiring context, one that is ultimately capable of provoking transformative experiences that lead to powerful social connections in the form of identity fusion. Borrowing tools from the free party scene to create a ritual environment could be beneficial to commercial retreat providers and those managing therapeutic CSP settings to try and provide ritualized environments for participants, which are more potent for meaningful, lasting social bonds and altruistic behavior.

To effectively evaluate the therapeutic value of psychedelics in the future, natural environments must be considered, as therapeutic drug consumption has been convincingly argued as inherently social (Kettner et al., 2021). Indeed, social networks and identities are considered integral to both physical and mental health more generally (Jetten et al., 2012). Compounding this, the plethora of psychedelic research has focused on individuals, leaving the intersubjective nature of altered states of consciousness and their pathway to shared experience and collective action less explored (Kettner et al., 2021).

### Limitations

This study suffers from several limitations. First, the 4Ds are used here as a pilot into investigating mechanisms for altered states of consciousness in natural populations. The items need to be replicated across different contexts and the dimensions need further investigation. Second, we are limited by the nature of our cross-sectional mediation analyses: while our results largely support our predictions, they cannot be considered to indicate causality, which would require experimentation or longitudinal analyses (Maxwell and Cole, 2007). Nonetheless, with bootstrapped analyses and careful interpretation of the indirect effects, our results could be taken as evidence of the paths proposed, especially when triangulated with further studies (Preacher and Hayes, 2004).

Third, due to our recruitment strategy, this study is faced with population differences. The effects were stronger in our more natural sample (recruited via snowball sampling on drug forums and rave social media) than the less natural sample of Prolific users. This may simply have been because Prolific users were significantly less likely to report attending illegal parties, and hence consumed fewer drugs, leading to lower scores on all subsequent outcome variables. Nonetheless, the relationships between key variables were consistent, even for the Prolific users. For instance, some Prolific users reported very low (disagree) awe responses, which still related to transformativeness, suggesting that even a small amount of awe has a (small) effect on transformativeness.

Fourth, the results rely on retrospective self-reports, from a self-selecting sample. The sample is also WEIRD (Western, Educated, Industrialized, Rich, and Democratic) due to its Northern hemisphere sample. Nonetheless, our research is perhaps best framed with the WILD paradigm, i.e., research that is Worldwide, *In Situ*, Local, and Distinct (Newson et al., 2020). In particular, the rave cultural context mirrors many of the core features of communal entheogenic psychedelic rituals. We are keen for future research to seek convergent validity for the 4Ds in more diverse contexts, especially other organic rituals that employ psychedelics, e.g., shamanic rituals.

Fifth, our data regarding drug use may suffer from the confounding effects of different combinations of poly-drug use (e.g., the concurrent use of psychedelics and cannabis or alcohol). In natural settings, poly-drug use is a major potential issue. We found that many participants combined one or more drugs with alcohol, but no clear pattern emerged to this. As Ives and Ghelani (2006) point out, reporting polydrug use is an important first step and we have made this data freely available on OSF. Furthermore, we did not disambiguate between MDMA and ‘pills’: although either may be ‘cut’ with other illegal or legal substances, this is typically the case for pills and their effects should not solely be attributed to MDMA. However, this reflects the real world and can be understood in a wider context of literature where psychedelic drugs are examined in controlled and laboratory settings.

Finally, our measure of ‘sharedness’ may have been too broad to accurately capture feelings of sharedness. Rather, the experience may have been with a smaller subset of the rave cohort, and bonding feelings then projected on to the wider group of ravers. Future research to quantify sharedness and disambiguate social targets is needed to disentangle this.

## Conclusion

Crowd rituals, like raves, are about ‘coming together’ and, in this capacity, rave offers something of an alternative to materialism. It has long been noted that the absence of meaning that characterizes modern consumer culture leads to its members feeling unfulfilled, from which mass experimentation and cultural and religious diversity ensue (Wallace, 1956). Rave is one expression of dissatisfaction with the current era and, for people who perceive the event as awe-inspiring, transmutes as a meaningful act rather than a temporary hedonistic escape from reality (Hutson, 2000:37). Both Durkheim and Turner regarded ritual as fundamental to sociocultural revitalization and, in this respect, rave involves processes at both individual and societal levels. There are myriad different forms of human ritual to match as many cultures and, as rave culture emerges, so too do its ritual practices to fulfill its members’ spiritual needs.

When physical connection is limited, e.g., during the COVID-19 pandemic, do we still have a need for physical proximity? How might the 4Ds and their associations with group synchrony help cohesion among young and vulnerable groups, who have been isolated during repeated lockdowns? What’s more, how might awe-inspiring, group experiences and physical closeness have a trickle-down effect to the rest of society? We found that while bonding to ravers was associated with transformative, awe-inspiring experiences fueled by the 4Ds, bonding to humanity was not – how might the boundary between bonding targets be stretched to encompass wider celebratory groups?

Elements of the 4Ds appear in large crowds – from sports fans to festival goers to political activists. We suspect that the 4Ds have the potential for a more prolific role in group bonding for close knit or therapeutic communities, where there is more potential for long term bonding in stable groups, compared to relatively anonymous large crowds at raves. Our model suggests that the 4Ds have a powerful effect in how events may be perceived as ritual and transformative, leading to meaningful bonds that are also associated with real-world, prosocial behaviors.

## Data Availability Statement

The datasets presented in this study can be found in online repositories. The names of the repository/repositories and accession number(s) can be found below: DOI 10.17605/OSF.IO/BZDE8.

## Ethics Statement

The studies involving human participants were reviewed and approved by University of Kent (004-ST-21). The patients/participants provided their informed consent to participate in this study.

## Author Contributions

MN and VM conceptualized the study, prepared the pre-registration, ran statistical analyses, and wrote the manuscript. MN, FC, and RK collected the data. RK and MN prepared the data. VM led statistical analyses, prepared the code, and ran the SEM. All authors discussed recruitment strategy and proofread the manuscript.

## Conflict of Interest

The authors declare that the research was conducted in the absence of any commercial or financial relationships that could be construed as a potential conflict of interest.

## Publisher’s Note

All claims expressed in this article are solely those of the authors and do not necessarily represent those of their affiliated organizations, or those of the publisher, the editors and the reviewers. Any product that may be evaluated in this article, or claim that may be made by its manufacturer, is not guaranteed or endorsed by the publisher.
