# Supplementary material for: ‘I Get High With a Little Help From My Friends’ - How Raves Can Invoke Identity Fusion and Lasting Co-operation via Transformative Experiences
Source: Front Psychol. 2021 Sep 24;12:719596. doi: 10.3389/fpsyg.2021.719596 (PMC8504457; doi:10.3389/fpsyg.2021.719596)
Supplement: Supplementary file 1 [file Data_Sheet_1.docx]

**‘I get high with a little help from my friends’**

Supplementary Materials

**Contents**

**Page Section**

| 2 | SM.1 Additional literature review (rave & liminality) |
| --- | --- |
| 3 | SM.2 Controlling for recruitment |
| 4 | SM.3 Method and materials |
| 5 | SM.4 Correlation matrix |
| 6-7 | SM.5 Full statistics for the mediation models of Hypothesis 1a and 1b |
| 8 | SM.6 Regression table for 4Ds and awe |
| 9-11 | SM.7 Separate regressions on AWE-S subscales |
| 12-15  16-17 | SM.8 Mediation model statistics and figure for Hypothesis 2a and 2b  SM.9 Full statistics for the mediation model of Hypothesis 3 |
| 18 | SM.10 Structural equation model statistics |
| 19 | SM.11 Exploratory analyses: gender |

**SM.1 Additional literature review**

***Liminal phases and the rave***

Gerard (2004:171) goes so far as to say that liminality is the root paradigm of rave culture, and it is with startling accuracy that the three stages of liminality described by van Gennep and Turner can be mapped on to rave (Gerard, 2004; Gauthier, 2004). The separation or pre-liminal phase can be seen in the choice of time for raves to start; nearly always post-dusk, so while the rest of society starts to sleep; initiates begin their journey. The choice in date for a party also helps to produce a liminal quality: though raves in the UK occur every weekend, especially throughout the summer months, there is a proliferation of events on the solstices, Halloween, New Year’s Eve and on the full moons, particularly for psy-trance or psychedelic trance parties. Van Gennep (1960) himself insisted that the celestial rites be included within the human rites, and it appears that the ambiguity produced by these liminal times is indeed reaped by rave organisers who regularly attune raves to the celestial calendar. Finally, the very location of raves gives away their rupturing quality: located on the edges of society, spatially separating initiates both physically and symbolically; common sites include dis-used airfields, woodlands, old quarries, underground bunkers, beaches, abandoned warehouses, squats or, for example, an eight storey dis-used sorting office for one particularly large ‘Scum-O-Ween' party.

The liminal phase is defined as transition to a sacred domain. Generosity and sharing are identified as binding strangers during rites of union in this phase (van Genep:28-30) and these behaviours are seen to permeate conventional boundaries, extending through social classes, race, ethnicity, genders, sexuality, and age. The strength of *communitas* to break structure and boundaries in rave culture has already been argued for over two decades (Tramacchi, 2000; St John, 2004) and has even been heralded as a spiritual healing for the youth (Hutson, 2000). An overarching anonymity and ambiguity amongst the *liminal personae* at a rave is achieved via a darkened environment, large crowd, similar clothing, and the homogenising effects of *communitas* is strongly felt by members of today’s individualistic, consumer society. Although not all people at raves take drugs, when they are consumed it is done so openly, certainly at unlicensed free parties. This may constitute a further mental split from customary everyday behaviour. The drugs themselves may well further contribute to a deepened liminal shift for the user as outlined above.

Finally, the reaggregation or post-liminal phase, returns the individual to a stable state in which they are expected to behave in accordance with customary norms (Turner, 1969). The social structure that has up until now been evaded is reinforced, and the transcendence into sacred is bracketed as separate and distinct from the profane. On reincorporation, the individual should be imbued with some of the sacred qualities or knowledge that was gained during the transcendental stage (Bloch, 1992). Ravers who have entered a liminal state thus have the potential to return to wider society with fresh or connected thinking, as the liminal nature of rave events encourages revitalisation through loss of identity and a merging with the collective body.

**SM.2 Controlling for recruitment**

To check that differences between the snowball and Prolific samples were not unduly biasing results, we ran a further series of models for the pre-registered hypotheses that included a binary recruitment type variable as a covariate (Table SM.1). These models were computed in SPSS v27, using the PROCESS macro v3.5 for moderation and mediation analyses (Hayes, 2017) so that a covariate could be easily added. The results were robust for H1a, H1c and H3. Significance became marginal for openness moderating the path from the 4Ds to transformativeness via awe in H1b and for one of the awe subscales in H1d. H2 was supported in terms of other ravers, but not humanity. In all models, recruitment type had significant effects on other variables (*p’s <* .005, except for bonding to humanity (*p* = .627).

**Table SM.1**

*Repeated pre-registered models adding recruitment type as a covariate*

| **Hypothesis** | **Original result** | **Result adding recruitment type as a covariate** |
| --- | --- | --- |
| **H1a** | Indirect path *b* = 0.17 [0.12, 0.23]. | Indirect path *b* = 0.23 [0.15, 0.31]. |
| **H1b** | Indirect path *b* = 0.05 [0.01, 0.10]; predicting the mediator *b* = 0.11 [0.02, 0.20]. | Indirect path *b* = .09 [-0.01, 0.18] ; predicting the mediator *b* = 0.10 [0.002, 0.195]. |
| **H1c** | Dance, sleep deprivation and drugs significantly predicted awe (*p’s <* .006), with no significant effect of drums (*p* = .68). | The effects of dance, sleep deprivation, and drugs were all significant in the expected direction (*p’s <* .004), and again with no effect of drums (*p* = .42). |
| **H1d** | Connection, vastness perception and physical sensations significantly predicted transformativeness (*p’s* < .010). Time perception, accommodation, and self diminishment were n.s. (*p’s* > .26). | Connection and vastness perception still had main effects (*p’s* < .006) but physical sensations was only marginally significant (*p* = .05). Time perception, accommodation, and self-diminishment were n.s. (*p’s >* .53). |
| **H2** | Ravers *b* = 0.11 [0.07, 0.16]; humanity *b* = -0.05 [-0.10, <-0.01]. | Ravers *b* = 0.08 [0.03, 0.14]; humanity *b* = -0.06 [-0.123, 0.001]. |
| **H3** | Ravers *b* = 0.05 [0.02, 0.09]; humanity = <-0.01 [-0.02, 0.01]. | Ravers *b* = 0.05 [0.003, 0.099]; humanity *b* = -0.02 [-0.063, 0.002]. |

**SM.3 Method and materials**

**Additional 4Ds** (excluded from analyses due to poor fit)

***Dancing:*** *How many hours did you dance for in total? (0-35h, 30m increments, label a whole day (18h)/ day and night (24h)*

***Drums:*** *How loud was the music? (60-130dB: (60 labelled normal conversation; 120 loud rock concert; motorcycle engine 95dB)*

***Sleep deprivation:*** *How long were you awake for, from the time you woke up prior to attending the rave, to the time you went to bed? (10-80h: label 24h/1 night, 48h/2 nights, 72h/3 nights)*

***Drugs:*** *For my body size, the dose I had was the minimum amount I would take to get high (0%) to the maximum I could personally take (100%) (1.43 increments so on a 0-70 scale)*

**SM. 4 Correlation matrix**

**Table SM.2**

*Means, standard deviations, and correlations with confidence intervals for main variables of interest*

| Variable | *M* | *SD* | 1 | 2 | 3 | 4 | 5 | 6 | 7 | 8 | 9 |
| --- | --- | --- | --- | --- | --- | --- | --- | --- | --- | --- | --- |
|  |  |  |  |  |  |  |  |  |  |  |  |
| 1. 4Ds | 5.13 | 0.97 |  |  |  |  |  |  |  |  |  |
|  |  |  |  |  |  |  |  |  |  |  |  |
| 2. Awe | 0.09 | 0.78 | .36** |  |  |  |  |  |  |  |  |
|  |  |  | [.29, .44] |  |  |  |  |  |  |  |  |
|  |  |  |  |  |  |  |  |  |  |  |  |
| 3. Transformativeness | -0.34 | 1.62 | .03 | .46** |  |  |  |  |  |  |  |
|  |  |  | [-.06, .11] | [.38, .52] |  |  |  |  |  |  |  |
|  |  |  |  |  |  |  |  |  |  |  |  |
| 4. Openness | 0.77 | 0.66 | .03 | .25** | .19** |  |  |  |  |  |  |
|  |  |  | [-.06, .11] | [.17, .33] | [.11, .27] |  |  |  |  |  |  |
|  |  |  |  |  |  |  |  |  |  |  |  |
| 5. Bonding with ravers | 3.17 | 1.18 | .24** | .39** | .36** | .20** |  |  |  |  |  |
|  |  |  | [.16, .32] | [.31, .46] | [.29, .44] | [.12, .28] |  |  |  |  |  |
|  |  |  |  |  |  |  |  |  |  |  |  |
| 6. Bonding with humanity | 3.23 | 1.16 | -.05 | -.10* | -.13** | .03 | .00 |  |  |  |  |
|  |  |  | [-.13, .04] | [-.18, -.01] | [-.21, -.04] | [-.05, .12] | [-.08, .09] |  |  |  |  |
|  |  |  |  |  |  |  |  |  |  |  |  |
| 7. Donation self | 3.17 | 3.72 | -.13** | -.18** | -.19** | -.15** | -.19** | -.06 |  |  |  |
|  |  |  | [-.22, -.05] | [-.26, -.09] | [-.28, -.11] | [-.23, -.06] | [-.27, -.11] | [-.15, .02] |  |  |  |
|  |  |  |  |  |  |  |  |  |  |  |  |
| 8. Donation rave charity | 2.29 | 2.93 | .09 | .22** | .30** | .19** | .25** | -.02 | -.48** |  |  |
|  |  |  | [-.00, .17] | [.14, .30] | [.22, .38] | [.11, .27] | [.17, .33] | [-.10, .07] | [-.55, -.41] |  |  |
|  |  |  |  |  |  |  |  |  |  |  |  |
| 9. Donation humanitarian charity | 4.55 | 3.45 | .07 | .00 | -.05 | -.00 | -.01 | .08 | -.67** | -.33** |  |
|  |  |  | [-.02, .16] | [-.09, .09] | [-.13, .04] | [-.09, .08] | [-.09, .08] | [-.00, .17] | [-.71, -.62] | [-.40, -.25] |  |
|  |  |  |  |  |  |  |  |  |  |  |  |
| 10. Sharedness | 2.83 | 0.73 | .09* | .33** | .38** | .07 | .35** | .01 | -.10* | .12** | .00 |
|  |  |  | [.00, .17] | [.25, .40] | [.30, .45] | [-.02, .15] | [.27, .42] | [-.08, .10] | [-.18, -.01] | [.03, .20] | [-.08, .09] |
|  |  |  |  |  |  |  |  |  |  |  |  |

*Note.* *M* and *SD* are used to represent mean and standard deviation, respectively. Values in square brackets indicate the 95% confidence interval for each correlation. The confidence interval is a plausible range of population correlations that could have caused the sample correlation (Cumming, 2014). * indicates *p* < .05. ** indicates *p* < .01.

**SM.5 Full statistics for the mediation models of Hypothesis 1a and 1b**

**Table SM.3**

*Model for Hypothesis 1a*

|  | **Estimate** | **SE** | **95% CI** | **z-value** | ***p*-value** |
| --- | --- | --- | --- | --- | --- |
| Transformativeness (Y) ~ |  |  |  |  |  |
| 4Ds (X; *Direct*) | -0.14 | 0.04 | [-0.23, -0.05] | -3.17 | 0.002 |
| Awe (M) | 0.50 | 0.04 | [0.42, 0.58] | 11.67 | <.001 |
| Awe (M) ~ |  |  |  |  |  |
| 4Ds (X) | 0.34 | 0.05 | [0.24, 0.43] | 7.12 | <.001 |
| *Indirect*  (X-M->Y) | 0.17 | 0.03 | [0.12, 0.23] | 5.94 | <.001 |
| Total  (*Direct + Indirect*) | 0.03 | 0.05 | [-0.08, 0.12] | 0.56 | 0.57 |
| R^2^ Transformativeness  R^2^ Awe | 0.22  0.11 |  |  |  |  |
| RMSEA | <0.001 |  |  |  |  |
| CFI | >0.999 |  |  |  |  |
| SRMR | <0.001 |  |  |  |  |

*Note*. All variables are standardised prior to the regression. Analysis bootstrapped (n=5000). Y=Outcome variable, X=Predictor variable, M=Mediator variable.

**Table SM.4**

*Model for Hypothesis 1b*

|  | **Estimate** | **SE** | **95% CI** | **z-value** | ***p*-value** |
| --- | --- | --- | --- | --- | --- |
| Transformativeness (Y) ~ |  |  |  |  |  |
| 4Ds (X; *Direct*) | -0.14 | 0.04 | [-0.23, -0.05] | -3.21 | 0.001 |
| Awe (M) | 0.50 | 0.04 | [0.41, 0.58] | 11.60 | <.001 |
| Awe (M) ~ |  |  |  |  |  |
| 4Ds (X) | 0.34 | 0.05 | [0.24, 0.43] | 7.03 | <.001 |
| Openness*4Ds (Mod) | 0.11 | 0.04 | [0.02, 0.20] | 2.39 | 0.02 |
| *Indirect1*  (X-M->Y) | 0.17 | 0.03 | [0.12, 0.23] | 5.82 | <.001 |
| *Indirect2*  (X-Mod->Y) | 0.05 | 0.02 | [0.01, 0.10] | 2.38 | 0.02 |
| Contrast  (*Indirect1* – *Indirect2*) | 0.11 | 0.03 | [0.05, 0.18] | 3.26 | 0.001 |
| Total1  (*Direct + Indirect1*) | 0.03 | 0.05 | [-0.07, 0.12] | 0.55 | 0.58 |
| Total2  (*Direct + Indirect2*) | -0.09 | 0.05 | [-0.19, 0.01] | -1.72 | 0.08 |
| R^2^ Transformativeness  R^2^ Awe | 0.22  0.13 |  |  |  |  |
| RMSEA | <0.001 |  |  |  |  |
| CFI | >0.999 |  |  |  |  |
| SRMR | <0.001 |  |  |  |  |

*Note*. All variables are standardised prior to the regression. Analysis bootstrapped (n=5000). Y=Outcome variable, X=Predictor variable, M=Mediator variable, Mod=Moderation.

**SM.6 Regression tables for 4Ds, awe, and personal transformativeness**

**Table SM.5**

*Regression predicting personal transformativeness with the 4Ds separately*

|  | **β** | **SE** | **95% CI** | ***t*-value** | ***p*-value** |
| --- | --- | --- | --- | --- | --- |
| Intercept | <0.01 | 0.04 | [-0.09, 0.09] | -0.03 | 0.98 |
| Dance | 0.17*** | 0.05 | [0.08, 0.26] | 3.57 | <.001 |
| Drums | -0.20*** | 0.05 | [-0.30, -0.11] | -4.28 | <.001 |
| Deprivation | -0.05 | 0.05 | [-0.15, 0.05] | -1.03 | 0.30 |
| Drugs | 0.12** | 0.05 | [0.03, 0.22] | 2.61 | 0.01 |
| Statistics | *F*(4,474)=8.459, *p* < .001 | | | | |
| Fit | *R*^2^ = .067**, 95% CI[.02,.11] | | | | |

*Note*. All variables were standardized before they were added to the regression. **p* < .05. ***p* < .01, ****p* < .001

**Table SM.6**

*Regression predicting awe by the 4Ds and the effect of having taken psychedelic drugs, MDMA, or both psychedelics and MDMA*

|  | **β** | **SE** | **95% CI** | ***t*-value** | ***p*-value** |
| --- | --- | --- | --- | --- | --- |
| Intercept | -0.12* | 0.05 | [-0.23, -0.01] | -2.21 | 0.03 |
| 4Ds | 0.28*** | 0.04 | [0.20, 0.37] | 6.29 | <.001 |
| Psychedelics | 0.59* | 0.24 | [0.12, 1.07] | 2.46 | 0.01 |
| MDMA | 0.30** | 0.10 | [0.10, 0.51] | 2.90 | 0.004 |
| Both | 0.28 | 0.16 | [-0.04, 0.59] | 1.74 | 0.09 |
| Statistics | F(4,476)=19.05, p < .001 | | | | |
| Fit | *R*^2^ = .138**, 95% CI[.08,.19] | | | | |

*Note*. All variables were standardized before they were added to the regression. **p* < .05. ***p* < .01, ****p* < .001

**SM.7 Separate regressions on AWE-S subscales**

**Table SM.7**

*Regression predicting* ***AWE-S Connection*** *with the 4Ds separately*

|  | **β** | **SE** | **95% CI** | ***t*-value** | ***p*-value** |
| --- | --- | --- | --- | --- | --- |
| Intercept | 0.00 | 0.04 | [-0.08, 0.08] | 0.00 | >0.99 |
| Dance | 0.25*** | 0.05 | [0.16, 0.34] | 5.41 | 0.00 |
| Drums | -0.11* | 0.05 | [-0.20, -0.01] | -2.29 | 0.02 |
| Deprivation | 0.03 | 0.05 | [-0.07, 0.12] | 0.54 | 0.59 |
| Drugs | 0.22*** | 0.05 | [0.13, 0.31] | 4.78 | 0.00 |
| Statistics | *F*(4,476)=17.44, *p* < .001 | | | | |
| Fit | *R*^2^ = .128**, 95% CI[.07,.18] | | | | |

**Table SM.8**

*Regression predicting* ***AWE-S Time perception*** *with the 4Ds separately*

|  | **β** | **SE** | **95% CI** | ***t*-value** | ***p*-value** |
| --- | --- | --- | --- | --- | --- |
| Intercept | 0.00 | 0.04 | [-0.08, 0.08] | 0.00 | >0.99 |
| Dance | 0.15*** | 0.05 | [0.06, 0.25] | 3.32 | 0.00 |
| Drums | 0.04 | 0.05 | [-0.05, 0.13] | 0.85 | 0.40 |
| Deprivation | 0.08 | 0.05 | [-0.01, 0.18] | 1.78 | 0.08 |
| Drugs | 0.21*** | 0.05 | [0.12, 0.30] | 4.47 | 0.00 |
| Statistics | *F*(4,476)=15.27, *p* < .001 | | | | |
| Fit | *R*^2^ = .114**, 95% CI[.06,.16] | | | | |

**Table SM.9**

*Regression predicting* ***AWE-S Vastness perception*** *with the 4Ds separately*

|  | **β** | **SE** | **95% CI** | ***t*-value** | ***p*-value** |
| --- | --- | --- | --- | --- | --- |
| Intercept | 0.00 | 0.04 | [-0.09, 0.09] | 0.00 | >0.99 |
| Dance | 0.22*** | 0.05 | [0.13, 0.32] | 4.68 | <.001 |
| Drums | -0.07 | 0.05 | [-0.16, 0.02] | -1.48 | 0.14 |
| Deprivation | 0.06 | 0.05 | [-0.04, 0.15] | 1.14 | 0.25 |
| Drugs | 0.12* | 0.05 | [0.02, 0.21] | 2.46 | 0.01 |
| Statistics | *F*(4,476)=10.06, *p* < .001 | | | | |
| Fit | *R*^2^ = .078**, 95% CI[.03,.12] | | | | |

**Table SM.10**

*Regression predicting* ***AWE-S Physical sensations*** *with the 4Ds separately*

|  | **β** | **SE** | **95% CI** | ***t*-value** | ***p*-value** |
| --- | --- | --- | --- | --- | --- |
| Intercept | 0.00 | 0.04 | [-0.09, 0.09] | 0.00 | >0.99 |
| Dance | 0.21*** | 0.05 | [0.11, 0.30] | 4.39 | <.001 |
| Drums | -0.03 | 0.05 | [-0.12, 0.07] | -0.54 | 0.59 |
| Deprivation | 0.05 | 0.05 | [-0.04, 0.14] | 1.03 | 0.31 |
| Drugs | 0.16*** | 0.05 | [0.06, 0.25] | 3.35 | <.001 |
| Statistics | *F*(4,476)=11.67, *p* < .001 | | | | |
| Fit | *R*^2^ = .089**, 95% CI[.04,.13] | | | | |

**Table SM.11**

*Regression predicting* ***AWE-S Need for accommodation*** *with the 4Ds separately*

|  | **β** | **SE** | **95% CI** | ***t*-value** | ***p*-value** |
| --- | --- | --- | --- | --- | --- |
| Intercept | 0.00 | 0.04 | [-0.09, 0.09] | 0.00 | >0.99 |
| Dance | 0.03 | 0.05 | [-0.06, 0.13] | 0.67 | 0.50 |
| Drums | 0.05 | 0.05 | [-0.04, 0.14] | 1.06 | 0.29 |
| Deprivation | 0.21*** | 0.05 | [0.11, 0.30] | 4.33 | <.001 |
| Drugs | 0.06 | 0.05 | [-0.03, 0.15] | 1.33 | 0.19 |
| Statistics | *F*(4,476)=9.14, *p* < .001 | | | | |
| Fit | *R*^2^ = .071**, 95% CI[.03,.11] | | | | |

**Table SM.12**

*Regression predicting* ***AWE-S Self-diminishment*** *with the 4Ds separately*

|  | **β** | **SE** | **95% CI** | ***t*-value** | ***p*-value** |
| --- | --- | --- | --- | --- | --- |
| Intercept | 0.00 | 0.04 | [-0.09, 0.09] | 0.00 | >0.99 |
| Dance | 0.11* | 0.05 | [0.01, 0.20] | 2.27 | 0.02 |
| Drums | 0.03 | 0.05 | [-0.07, 0.12] | 0.58 | 0.56 |
| Deprivation | 0.17*** | 0.05 | [0.07, 0.26] | 3.51 | <.001 |
| Drugs | 0.08 | 0.05 | [-0.01, 0.18] | 1.80 | 0.07 |
| Statistics | *F*(4,476)=9.711, *p* < .001 | | | | |
| Fit | *R*^2^ = .075**, 95% CI[.03,.12] | | | | |

**SM.8 Mediation model statistics and figure for Hypothesis 2a and 2b**

**Table SM.13**

*Full statistics for mediation model for Hypothesis 2a*

|  | **Estimate** | **SE** | **95% CI** | **z-value** | ***p*-value** |
| --- | --- | --- | --- | --- | --- |
| Tranformativeness (M) ~ |  |  |  |  |  |
| Awe (X) | 0.46 | 0.04 | [0.38, 0.53] | 11.45 | <.001 |
| Bonding with ravers (Y1) ~ |  |  |  |  |  |
| Awe (X; *Direct*) | 0.24 | 0.05 | [0.14, 0.34] | 4.79 | <.001 |
| Tranformativeness (M) | 0.26 | 0.05 | [0.16, 0.35] | 5.39 | <.001 |
| Bonding with humanity (Y2) ~ |  |  |  |  |  |
| Awe (X; *Direct*) | -0.11 | 0.05 | [-0.21, <-0.01] | -1.98 | 0.05 |
| Tranformativeness (M) | -0.08 | 0.05 | [-0.19, 0.02] | -1.56 | 0.12 |
| Bonding with ravers (Y1) ~~  Bonding with humanity (Y2) | 0.04 | 0.04 | [-0.04, 0.12] | 0.87 | 0.39 |
| *Indirect 1*  (X-M->Y1) | 0.11 | 0.02 | [0.07, 0.16] | 4.53 | <.001 |
| *Indirect 1*  (X-M->Y2) | -0.05 | 0.02 | [-0.10, <-0.01] | -1.98 | 0.05 |
| Total 1  (*Direct + Indirect1*) | 0.37 | 0.04 | [0.29, 0.45] | 8.85 | <.001 |
| Total 2  (*Direct + Indirect2*) | -0.13 | 0.05 | [-0.22, -0.04] | -2.85 | <.001 |
| R^2^ Tranformativeness | 0.20 |  |  |  |  |
| R^2^ Bonding with ravers | 0.18 |  |  |  |  |
| R^2^ Bonding with humanity | 0.03 |  |  |  |  |
| RMSEA | <0.001 |  |  |  |  |
| CFI | >0.999 |  |  |  |  |
| SRMR | <0.001 |  |  |  |  |

*Note*. All variables are standardised prior to the regression. Analysis bootstrapped (n=5000). Y’s=Outcome variables, X=Predictor variable, M=Mediator variable.


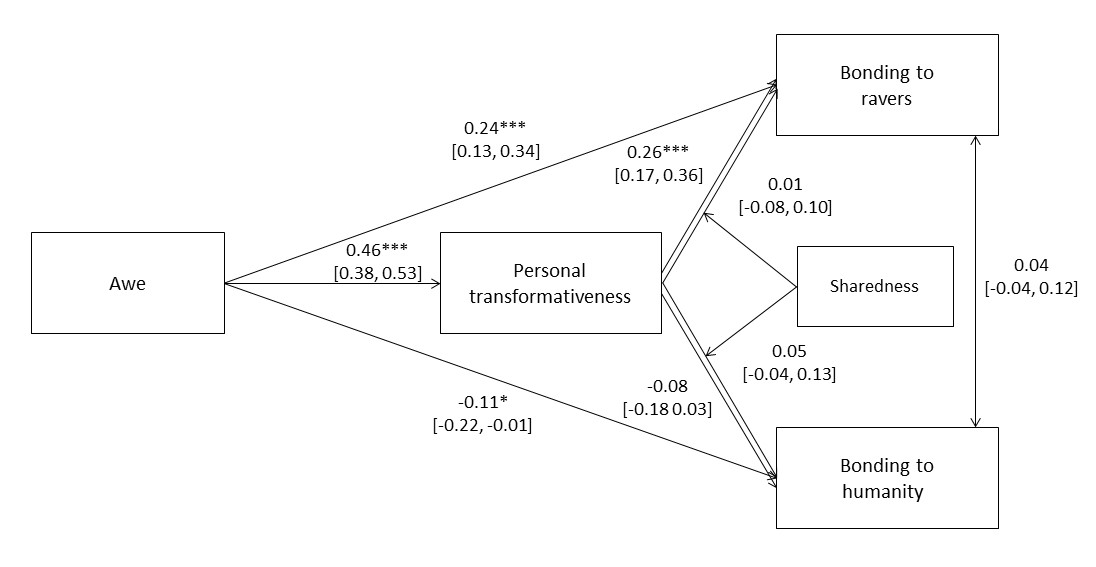


*Figure SM.1* Mediation model predicting bonding to ravers and humanity from awe via personal transformativeness with sharedness as moderator

**Table SM.14**

*Full statistics for mediation model for Hypothesis 2b*

|  | **Estimate** | **SE** | **95% CI** | **z-value** | ***p*-value** |
| --- | --- | --- | --- | --- | --- |
| Tranformativeness (M) ~ |  |  |  |  |  |
| Awe (X) | 0.46 | 0.04 | [0.38, 0.53] | 11.37 | <.001 |
| Bonding with ravers (Y1) ~ |  |  |  |  |  |
| Awe (X; *Direct*) | 0.24 | 0.05 | [0.13, 0.34] | 4.63 | <.001 |
| Tranformativeness (M) | 0.26 | 0.05 | [0.17, 0.36] | 5.58 | <.001 |
| Transformativeness*Sharedness  (Mod) | 0.01 | 0.04 | [-0.08, 0.10] | 0.20 | 0.84 |
| Bonding with humanity (Y2) ~ |  |  |  |  |  |
| Awe (X; *Direct*) | -0.11 | 0.05 | [-0.22, -0.01] | -2.13 | 0.03 |
| Tranformativeness (M) | -0.08 | 0.05 | [-0.18, 0.03] | -1.48 | 0.14 |
| Transformativeness*Sharedness  (Mod) | 0.05 | 0.04 | [-0.04, 0.13] | 1.10 | 0.27 |
| Bonding with ravers (Y1) ~~  Bonding with humanity (Y2) | 0.04 | 0.04 | [-0.04, 0.12] | 0.87 | 0.39 |
| *Indirect 1*  (X-M->Y1) | 0.11 | 0.03 | [0.06, 0.16] | 4.41 | <.001 |
| *Indirect 1*  (X-M->Y2) | -0.05 | 0.02 | [-0.10, <-0.01] | -2.14 | 0.03 |
| *Indirect 3*  (X-Mod->Y1) | <0.01 | 0.02 | [-0.04, 0.04] | 0.20 | 0.84 |
| *Indirect 4*  (X-Mod->Y2) | 0.02 | 0.02 | [-0.02, 0.06] | 1.09 | 0.28 |
| Contrast 1  (*Indirect1*- *Indirect3*) | 0.11 | 0.04 | [0.04, 0.18] | 2.99 | 0.00 |
| Contrast 2  (*Indirect1*2 - *Indirect4*) | -0.07 | 0.04 | [-0.14, -0.01] | -2.11 | 0.04 |
| Total 1  (*Direct + Indirect1*) | 0.37 | 0.04 | [0.29, 0.45] | 8.92 | <.001 |
| Total 2  (*Direct + Indirect2*) | -0.13 | 0.05 | [-0.22, -0.04] | -2.79 | 0.01 |
| R^2^ Tranformativeness | 0.20 |  |  |  |  |
| R^2^ Bonding with ravers | 0.18 |  |  |  |  |
| R^2^ Bonding with humanity | 0.03 |  |  |  |  |
| RMSEA | <0.001 |  |  |  |  |
| CFI | >0.999 |  |  |  |  |
| SRMR | <0.001 |  |  |  |  |

*Note*. All variables are standardised prior to the regression. Analysis bootstrapped (n=5000). X=Predictor variable, M=Mediator variable, Mod=Moderator variable, Y’s=Outcome variables**.**

**Table SM.15**

*Regression predicting bonding with ravers with the awe subscales*

|  | **β** | **SE** | **95% CI** | ***t*-value** | ***p*-value** |
| --- | --- | --- | --- | --- | --- |
| Intercept | -0.25*** | 0.05 | [-0.35, -0.16] | -5.12 | <.001 |
| Connection | 0.31*** | 0.06 | [0.19, 0.42] | 5.24 | <.001 |
| Time perception | <0.01 | 0.05 | [-0.10, 0.11] | 0.07 | 0.95 |
| Vastness perception | 0.13 | 0.07 | [<-0.01, 0.26] | 1.94 | 0.05 |
| Physical | 0.22*** | 0.05 | [0.12, 0.33] | 4.11 | <.001 |
| Accommodation | -0.24*** | 0.06 | [-0.35, -0.12] | -4.15 | <.001 |
| Self-diminishment | -0.06 | 0.05 | [-0.16, 0.03] | -1.28 | 0.20 |
| Statistics | *F*(6,474)=32.19, *p* < .001 | | | | |
| Fit | *R*^2^ = .290**, 95% CI[.22,34] | | | | |

*Note*. All variables were standardized before they were added to the regression. **p* < .05. ***p* < .01, ****p* < .001

The regression predicting bonding with humanity with awe subscales was not significant (*F*(6,474)=1.854, *p* = 0.09; *R*^2^ = .023, 95% CI[.00, .04]), and as such will not be reported further.

**SM.9 Full statistics for the mediation model of Hypothesis 3**

**Table SM.16**

*Full statistics for mediation model of Hypothesis 3*

|  | **Estimate** | **SE** | **95% CI** | **z-value** | ***p*-value** |
| --- | --- | --- | --- | --- | --- |
| Bonding with ravers (M1) ~ |  |  |  |  |  |
| Tranformativeness (X) | 0.36 | 0.04 | [0.27, 0.44] | 8.02 | <.001 |
| Bonding with humanity (M2) ~ |  |  |  |  |  |
| Tranformativeness (X) | -0.16 | 0.05 | [-0.24, -0.06] | -3.28 | 0.001 |
| Donation rave charity (Y1) ~ |  |  |  |  |  |
| Tranformativeness (X; *Direct*) | 0.26 | 0.05 | [0.15, 0.36] | 4.87 | <.001 |
| Bonding with ravers (M1) | 0.15 | 0.05 | [0.06, 0.25] | 3.07 | 0.002 |
| Bonding with humanity (M2) | 0.02 | 0.05 | [-0.08, 0.11] | 0.32 | 0.75 |
| Donation humanitarian charity (Y2) ~ |  |  |  |  |  |
| Tranformativeness (X; *Direct*) | -0.05 | 0.05 | [-0.15, 0.05] | -1.02 | 0.31 |
| Bonding with ravers (M1) | <0.01 | 0.05 | [-0.10, 0.10] | 0.04 | 0.97 |
| Bonding with humanity (M2) | 0.08 | 0.05 | [-0.01, 0.17] | 1.59 | 0.11 |
| Bonding with ravers (M1) ~~  Bonding with humanity (M2) | 0.02 | 0.04 | [-0.07, 0.10] | 0.37 | 0.71 |
| *Indirect 1*  (X-M1->Y1) | 0.05 | 0.02 | [0.02, 0.10] | 2.89 | 0.004 |
| *Indirect 1*  (X-M1->Y2) | 0.01 | 0.02 | [-0.03, 0.04] | 0.32 | 0.75 |
| *Indirect 3*  (X-M2->Y1) | <0.01 | 0.01 | [-0.02, 0.02] | -0.04 | 0.97 |
| *Indirect 4*  (X-M2->Y2) | -0.01 | 0.01 | [-0.04, <0.01] | -1.34 | 0.18 |
| Contrast 1  (*Indirect1*- *Indirect3*) | 0.05 | 0.03 | [<-0.01, 0.11] | 1.78 | 0.08 |
| Contrast 2  (*Indirect1*2 - *Indirect4*) | 0.01 | 0.01 | [-0.01, 0.05] | 0.90 | 0.37 |
| Total 1  (*Direct + Indirect1*) | 0.31 | 0.05 | [0.21, 0.41] | 6.46 | <.001 |
| Total 2  (*Direct + Indirect2*) | -0.05 | 0.05 | [-0.15, 0.04] | -1.07 | 0.29 |
| R^2^ Bonding with ravers | 0.13 |  |  |  |  |
| R^2^ Bonding with humanity | 0.02 |  |  |  |  |
| R^2^ Donation rave charity | 0.12 |  |  |  |  |
| R^2^ Donation humanitarian charity | 0.01 |  |  |  |  |
| RMSEA | <0.001 |  |  |  |  |
| CFI | >0.999 |  |  |  |  |
| SRMR | <0.001 |  |  |  |  |

*Note*. All variables are standardised prior to the regression. Analysis bootstrapped (n=5000). X=Predictor variable, M’s=Mediator variables, Y’s=Outcome variables**.**

**SM.10 Structural equation model statistics**

**Table SM.17**

*Full structural equation model of the rave pathway*

|  | **Estimate** | **SE** | **95% CI** | **z-value** | ***p*-value** |
| --- | --- | --- | --- | --- | --- |
| Awe (M1/X2) ~ |  |  |  |  |  |
| 4Ds (X1) | 0.34 | 0.05 | [0.25, 0.43] | 7.30 | <.001 |
| Openness*4Ds (Mod) | 0.11 | 0.05 | [0.02, 0.2] | 2.41 | 0.02 |
| Transformativeness (Y1/M2) ~ |  |  |  |  |  |
| 4Ds (X1) | -0.15 | 0.04 | [-0.23, -0.06] | -3.41 | <.001 |
| Awe (M1) | 0.51 | 0.04 | [0.42, 0.59] | 12.16 | <.001 |
| Bonding with ravers (Y2/M3) ~ |  |  |  |  | <.001 |
| Awe (M1/X2) | 0.25 | 0.05 | [0.16, 0.35] | 5.21 | <.001 |
| Transformativeness (Y1/M2) | 0.24 | 0.05 | [0.14, 0.34] | 4.66 | <.001 |
| Donation self (Y3) ~ |  |  |  |  |  |
| Bonding with ravers (Y2/M3) | -0.12 | 0.05 | [-0.22, -0.03] | -2.49 | 0.01 |
| Transformativeness (Y1/M2) | -0.14 | 0.05 | [-0.24, -0.05] | -2.99 | <.001 |
| Donation rave charity (Y4) ~ |  |  |  |  |  |
| Bonding with ravers (Y2/M3) | 0.15 | 0.05 | [0.06, 0.25] | 3.07 | <.001 |
| Transformativeness (Y1/M2) | 0.26 | 0.05 | [0.15, 0.36] | 4.84 | <.001 |
| R^2^ Awe | 0.14 |  |  |  |  |
| R^2^ Transformativeness | 0.23 |  |  |  |  |
| R^2^ Bonding with ravers | 0.18 |  |  |  |  |
| R^2^ Donation self | 0.05 |  |  |  |  |
| R^2^ Donation rave charity | 0.12 |  |  |  |  |

Note. All variables were standardised prior to analysis. Analysis bootstrapped n=5000.

**SM.11 Exploratory analyses: gender**

**Table SM.18**

*Gender differences in key variables*

|  | **Gender** | ***N*** | ***M (SD)*** | ***t*** | ***df*** | ***p*** | **95% Confidence Interval of the Difference** |
| --- | --- | --- | --- | --- | --- | --- | --- |
| 4Ds (Dance) | M | 208 | 5.69 (1.33) | -2.26 | 403.88 | .024 | -0.49; -0.03 |
|  | F | 263 | 5.95 (1.12) |  |  |  |  |
| 4Ds (Drugs) | M | 208 | 4.67 (1.58) | 2.78 | 469 | .006 | 0.12, 0.72 |
|  | F | 263 | 4.25 (1.66) |  |  |  |  |
| Openness | M | 208 | 0.85 (0.63) | 2.948 | 469 | .003* | 0.06; 0.30 |
|  | F | 263 | 0.67 (0.69) |  |  |  |  |
| Transformativeness | M | 206 | -0.06 (1.52) | 3.44 | 455.8 | .001* | 0.22; 0.80 |
|  | F | 263 | -0.57 (1.67) |  |  |  |  |
| Bonding (ravers) | M | 208 | 3.31 (1.14) | 2.04 | 469 | .042 | 0.01; 0.43 |
|  | F | 263 | 3.10 (1.16) |  |  |  |  |
| Drums, sleep deprivation, awe and donations (rave) were not statistically different, *p*'s > .159  * Bonferroni-corrected significance, *p* < .006   \|  \| **Gender** \| **Reported 'yes'** \| ***χ2*** \| ***p*** \| \| --- \| --- \| --- \| --- \| --- \| \| Illegal event \| M \| 36.10% \| 11.91 \| .001* \| \|  \| F \| 21.70% \|  \|  \| \| Took drugs \| M \| 57.70% \| 25.9 \| <.001* \| \|  \| F \| 34.20% \|  \|  \| \| Psychedelics \| M \| 16.40% \| 7.99 \| .005* \| \|  \| F \| 8.00% \|  \|  \| \| MDMA \| M \| 40.10% \| 4.41 \| .036 \| \|  \| F \| 30.80% \|  \|  \|   * Bonferroni-corrected significance, *p* < .013 | | | | | | | |
|  | | | |  |  |  |  |
